# Assessing Anti-HCMV Cell Mediated Immune Responses in Transplant Recipients and Healthy Controls Using a Novel Functional Assay

**DOI:** 10.3389/fcimb.2020.00275

**Published:** 2020-06-26

**Authors:** Charlotte J. Houldcroft, Sarah E. Jackson, Eleanor Y. Lim, George X. Sedikides, Emma L. Davies, Claire Atkinson, Megan McIntosh, Ester B. M. Remmerswaal, Georgina Okecha, Frederike J. Bemelman, Richard J. Stanton, Matthew Reeves, Mark R. Wills

**Affiliations:** ^1^Department of Medicine, Addenbrookes Hospital, University of Cambridge, Cambridge, United Kingdom; ^2^Division of Infection and Immunity, Institute for Immunity and Transplantation, University College London, London, United Kingdom; ^3^Department of Experimental Immunology, Amsterdam Infection and Immunity Institute, Amsterdam UMC, University of Amsterdam, Amsterdam, Netherlands; ^4^Renal Transplant Unit, Division of Internal Medicine, Academic Medical Centre, Amsterdam UMC, University of Amsterdam, Amsterdam, Netherlands; ^5^Division of Infection and Immunity, School of Medicine, Cardiff University, Cardiff, United Kingdom

**Keywords:** herpesvirus, host-pathogen interactions, secreted immunity, T cells, transplantation, cell-mediated immunity, antiviral, cytomegalovirus

## Abstract

HCMV infection, reinfection or reactivation occurs in 60% of untreated solid organ transplant (SOT) recipients. Current clinical approaches to HCMV management include pre-emptive and prophylactic antiviral treatment strategies. The introduction of immune monitoring to better stratify patients at risk of viraemia and HCMV mediated disease could improve clinical management. Current approaches quantify T cell IFNγ responses specific for predominantly IE and pp65 proteins *ex vivo*, as a proxy for functional control of HCMV *in vivo*. However, these approaches have only a limited predictive ability. We measured the IFNγ T cell responses to an expanded panel of overlapping peptide pools specific for immunodominant HCMV proteins IE1/2, pp65, pp71, gB, UL144, and US3 in a cohort of D+R– kidney transplant recipients in a longitudinal analysis. Even with this increased antigen diversity, the results show that while all patients had detectable T cell responses, this did not correlate with control of HCMV replication in some. We wished to develop an assay that could directly measure anti-HCMV cell-mediated immunity. We evaluated three approaches, stimulation of PBMC with (i) whole HCMV lysate or (ii) a defined panel of immunodominant HCMV peptides, or (iii) fully autologous infected cells co-cultured with PBMC or isolated CD8^+^ T cells or NK cells. Stimulation with HCMV lysate often generated non-specific antiviral responses while stimulation with immunodominant HCMV peptide pools produced responses which were not necessarily antiviral despite strong IFNγ production. We demonstrated that IFNγ was only a minor component of secreted antiviral activity. Finally, we used an antiviral assay system to measure the effect of whole PBMC, and isolated CD8^+^ T cells and NK cells to control HCMV in infected autologous dermal fibroblasts. The results show that both PBMC and especially CD8^+^ T cells from HCMV seropositive donors have highly specific antiviral activity against HCMV. In addition, we were able to show that NK cells were also antiviral, but the level of this control was highly variable between donors and not dependant on HCMV seropositivity. Using this approach, we show that non-viraemic D+R+ SOT recipients had significant and specific antiviral activity against HCMV.

## Introduction

Human cytomegalovirus (HCMV) remains a significant cause of mortality and morbidity in adult and pediatric solid organ (Razonable, 2005) and hematopoietic stem cell (Hiwarkar et al., 2013) transplant recipients. Viraemia in solid organ transplant (SOT) recipients can result from a primary infection, reinfection, superinfection with multiple strains (Görzer et al., 2010; Cudini et al., 2019), or from reactivation of the host's own HCMV strain(s) (Atabani et al., 2012). HCMV viraemia and disease in SOT recipients is associated with a number of risk factors, including organ type (with larger transfers of lymphoid tissue conveying higher risk, due to the larger latent virus load present) (Razonable and Humar, 2013), the degree of immune suppression (required to prevent organ rejection) the SOT recipient is receiving, as well as other co-infections and co-morbidities and relative HCMV serostatus (Razonable, 2005; Atabani et al., 2012).

Relative HCMV serostatus can be used to divide solid organ donors and recipients into four groups with distinct HCMV viraemia and disease risk profiles. Donor seronegative, recipient seronegative (D–R–) solid organ transplants have the lowest likelihood of HCMV infection or disease. Donor seronegative, recipient seropositive (D–R+) transplants have a low risk of viraemia, as the recipient has pre-existing cellular immunity to HCMV and no exogenous HCMV strains are introduced by the donor organ; HCMV viraemia comes from reactivation of the recipient's own virus(es). Donor seropositive, recipient seropositive transplants (D+R+) have an intermediate risk of viraemia, because while the recipient has pre-existing immunity, HCMV re-infection or superinfection by donor strains may occur, as well as reactivation of the recipient's own HCMV. The highest risk of HCMV viraemia and disease is seen in donor seropositive, recipient seronegative (D+R–) transplants. In this situation, the donor organ can transmit HCMV to the immunologically HCMV-naïve recipient, causing primary infection with one or more HCMV strains (Atabani et al., 2012).

Antiviral prophylaxis and pre-emptive treatment are important tools for the prevention and management of HCMV disease in immune suppressed populations (Lumley et al., 2019). Using a prophylactic treatment strategy, all patients considered at risk of HCMV viraemia and disease receive antiviral treatment for a defined period of time immediately post-transplant. In contrast, pre-emptive treatment monitors asymptomatic patients for evidence of HCMV replication [DNAemia measured by quantitative nucleic acid testing (QNAT)] and treats with antivirals once a threshold is reached (Razonable and Humar, 2019). In clinical practice, this means that pre-emptive treatment requires frequent (e.g., twice-weekly) monitoring of HCMV DNA in blood, and commencing antiviral treatment at a defined DNAemia threshold, for example 2,520 IU/ml (Griffiths et al., 2016). Recent data suggest that the prophylactic strategy requires less intensive patient monitoring in SOT, and is associated with lower viraemia and a lower risk of HCMV reactivation/reinfection compared to pre-emptive treatment (Griffiths, 2019). However prophylactic treatment also leads to an increased risk of late-onset disease due to poorer cell-mediated immunity in D+R– transplants (Limaye et al., 2019); and poorer patient outcomes, including reduced kidney function and increased risk of graft rejection (Blazquez-Navarro et al., 2019). There is conflicting evidence as to whether prophylaxis significantly increases the risk of drug resistance (Hakki and Chou, 2011; López-Aladid et al., 2017). However, it is notable that HCMV viraemia and disease only seems to occur in a subset of these “at-risk” individuals.

The occurrence of HCMV viraemia and disease in patient groups with T cell immune deficiencies (untreated HIV/AIDS, inborn errors of immunity) or suppression (SOT recipients) highlights the importance of cell-mediated immunity (CMI) in the control of HCMV (Fiala et al., 1986; Bowen et al., 1997; Bunde et al., 2005; Gerna et al., 2006; McLaughlin et al., 2017). The practice of clinical monitoring for development of HCMV-specific immunity using HCMV-specific interferon gamma (IFNγ) production has been studied in both solid organ and hematopoietic stem cell transplant recipients and has gained traction in recent years. In solid organ transplant recipients, the utility of such an approach is in assessing the risk of late-onset HCMV disease after cessation of prophylaxis in high-risk (D+R–) transplant recipients, predicting seropositive (R+) patients who may spontaneously clear HCMV infection (Kumar et al., 2018), and predicting risk of relapse of HCMV viraemia or disease (Haidar et al., 2020). In contrast, the predictive power of CMI assays was much lower in high-risk seronegative (R–) SOT recipients (Kumar et al., 2018; Haidar et al., 2020), where an effective assay might yield the greatest benefit. In the hematopoietic stem cell transplant population, immune monitoring is used with pre-emptive treatment strategies and can be used to predict early or recurrent reactivation, to shorten duration of anti-HCMV therapy, and to predict individuals likely to clear HCMV infection spontaneously (Yong et al., 2018). The main advantage offered with immune monitoring is to allow clinicians to tailor the use of antiviral therapies, thereby reducing the attendant therapeutic complications, such as myelosuppression with ganciclovir/valganciclovir, and electrolyte imbalances with foscarnet. Better immune monitoring would also allow for the preparation of e.g., HCMV-specific therapeutic T cells in patients thought highly likely to fail antiviral therapy (Neuenhahn et al., 2017). This group would include patients with pre-existing antiviral resistance mutations or patients infected with more than one HCMV strain (Coaquette et al., 2004; Lisboa et al., 2011). These factors may overlap with and be exacerbated by poor cell-mediated antiviral immunity. However, the limitations of immune monitoring, such as high costs, slow turnaround times, and lack of standardization remain to be addressed (Haidar et al., 2020).

There are a number of assays currently used to provide an *ex vivo* measure of HCMV-specific cellular immunity. Immune monitoring assays can be broadly placed in to four groups. EliSpot-based assays, such as T-Spot (Kumar et al., 2018) and T-Track (Banas et al., 2017), use peptides from pp65 and IE1/2 and enumerate CD4^+^ and CD8^+^ T cell responses. However, individuals responding to epitopes from other HCMV proteins would not be covered by these assays, although the assay is HLA-agnostic. While EliSpot-based assays can be adapted to analyse a wider range of antigens (whether with HCMV lysate or peptide pools), this approach is more often used for research than clinical assays (e.g., Mohty et al., 2004; Goodell et al., 2007; Jackson et al., 2017b). There are also ELISA-based assays, such as QuantiFERON-CMV (Qiagen) (Walker et al., 2007). QuantiFERON-CMV measures CD8^+^ T cell responses to 22 defined epitopes from IE1 and 2, pp28, pp50, pp65, and gB with restricted HLA coverage, and may be confounded by lymphopenia (Giulieri and Manuel, 2011). MHC class I HCMV tetramer/multimer peptide complex staining (Yong et al., 2018) allows the detection and quantification of HCMV-specific cytotoxic CD8^+^ T cells, covering known epitopes in pp50, pp65, and IE1 (Borchers et al., 2011). These HCMV-specific CTLs are associated with protection from viraemia in some patient populations, although not currently considered predictive (Kotton et al., 2018). Flow cytometry-based intracellular cytokine staining is also used for research applications, but is not as widely used for diagnostic purposes (Fernández-Ruiz et al., 2018) because of the requirement for flow cytometry equipment and expertise (Rogers et al., 2020), despite its potential to predict both viraemia and disease (Kotton et al., 2018). Most non-flow cytometry-based approaches are restricted to peptides recognized specifically by HLA types more common in populations of European descent. More generally, these assays are measuring the ability of a T cell to respond to an antigen and using that as a correlate of inferred antiviral activity.

The majority of these HCMV-immune monitoring assays, and particularly the EliSpot/FluoroSpot and ELISA-based assays, focus on production of a single cytokine in response to HCMV—IFNγ. There are problems with both the negative and positive predictive value of these assays (Chanouzas et al., 2018; Deborska-Materkowska et al., 2018; Jarque et al., 2018; Fernández-Ruiz et al., 2020); while other prospective studies have found positive IFNγ EliSpot responses to be predictive of protection against HCMV viraemia or disease necessitating a change in treatment strategy (Kumar et al., 2019). IFNγ responses to HCMV as measured by ELISA and EliSpot are clearly measuring part—but not all—of HCMV CMI, because viraemia can occur in the presence of IFNγ responses to HCMV; and viraemia does not necessarily occur in the absence of IFNγ responses to HCMV. As such it is likely that other secreted and cell-mediated factors are involved, including CMI responses to epitopes not included in most commercial assays; other cytokines with antiviral activity; the responses of other arms of the immune system beyond CD8^+^ T cells [e.g., CD4^+^ T cells (Watkins et al., 2012); NK cells (Venema et al., 1994); monocyte-derived macrophages (Becker et al., 2018); γδ T cells (Knight et al., 2010; Kaminski et al., 2016); antibodies (Baraniak et al., 2018)]; and host and viral genetic variation (Sezgin et al., 2019; Suárez et al., 2019).

In this study we have examined by FluoroSpot the IFNγ response to overlapping peptides from a much broader range of immunodominant HCMV proteins in D+R– kidney transplant recipients experiencing primary HCMV infection, correlated with patient DNAemia over a time course post-transplantation. These results show that detection of HCMV-specific T cells at frequencies similar to normal healthy controls was not predictive of the ability to control episodes of viraemia. We have also studied the antiviral activity of supernatants derived from PBMC stimulated with HCMV-infected cell lysate as well as immunodominant peptide pools in a virus dissemination assay system. Using this system, we demonstrated that lysate and peptide stimulation of PBMC are imperfect ways to measure HCMV secreted antiviral immunity, as many donors reacted non-specifically to lysate stimulation or did not produce antiviral responses to peptide stimulation. Finally, we utilized a fully autologous virus dissemination assay co-cultured with whole PBMC, CD8^+^ T cells, or NK cells to determine the antiviral capacity of these immune effectors against HCMV-infected fibroblasts. In healthy donors both whole PBMC and isolated CD8^+^ T cells were highly effective in the control of HCMV replication. The results of the NK cell co-cultures show that while some donors were able to control HCMV replication, other donors had much poorer antiviral activity. We then demonstrate that PBMC and CD8^+^ T cells derived from D+R+ kidney transplant recipients who control their viraemia post-transplant, could control HCMV dissemination similarly to an immunocompetent, healthy HCMV seropositive individual. Our data lead us to conclude that autologous cell-mediated assays are the most powerful way to characterize the functionality of the antiviral immune response to HCMV. Importantly, this approach will allow us to stratify patients based on the ability of their CMI to control HCMV *ex vivo* and, furthermore, could have important implications for our understanding of the key elements of the immune response which are important for the control of HCMV.

## Materials and Methods

### Recruitment: Kidney Transplant Recipients

Seven seropositive donor to seronegative recipient (D+R–) kidney transplant patients were recruited by Academisch Medisch Centrum (AMC), Amsterdam, who experienced primary HCMV infection post-transplantation. Transplants took place between 2003 and 2009. Ethical permission was granted by the Medical Ethics Committee of the AMC, Amsterdam and all patients gave informed written consent in accordance with the Declaration of Helsinki. Donor and recipient serostatus were defined using a microparticle enzyme immunoassay as previously described (Remmerswaal et al., 2012). PBMC were collected at multiple time points after transplantation, with subsequent samples collected at varying time points up to a maximum of 158 weeks post-transplantation, isolated by density centrifugation and cryopreserved (Remmerswaal et al., 2012). Virus load monitoring was performed by quantitative PCR (qPCR) as previously described (Boom et al., 1999). PBMC were a kind gift from Professors I. J. M. ten Berge and R. A. W. van Lier (Amsterdam Renal Transplant Unit).

Four seropositive donor to seropositive recipient (D+R+) kidney transplant patients were recruited by the Royal Free Hospital, London. Ethical permission for “UCL17-0008 Analysis of Cytomegalovirus Pathogenesis in Solid Organ Transplant Patients Study” was granted by the London—Queen Square Research Ethics Committee (REC reference 17/LO/0916). Informed written consent was obtained from all patients included in this study prior to providing pseudo-anonymised research samples (blood, urine, saliva, skin, and bile). No patients in the UCL cohort received antiviral therapy as they did not develop detectable DNAemia, following previously published treatment guidelines (Griffiths et al., 2016). Virus load (DNAemia) monitoring was performed by qPCR as previously described (Mattes et al., 2005; Atabani et al., 2012).

For all donors used in this study, PBMC derived from blood samples were collected and stored in a cell bank for subsequent analysis. The study was designed in this way for two reasons, firstly because of the time required to grow out a human dermal fibroblast line (weeks) which are required for the autologous viral dissemination assays. Secondly, the assays developed in these studies are to be used in a longitudinal analysis of D+R– patients post-transplantation to understand which immune responses confer protection from viraemia. Blood samples collected for PBMC isolation and determination of viraemia will be taken at regular time points post-transplantation so that PBMC from time points with and without viraemia could be assayed at multiple time points in parallel. T cell responses from normal healthy volunteers were thus treated in same way. Our freezing and thawing protocols are defined and consistent, and where appropriate an anti CD3/CD28 positive control is included, such that donors that failure to respond to polyclonal stimulation are excluded from analysis.

Patients are summarized in Table 1 and Supplementary Table 1 (D+R–) and Supplementary Table 2 (D+R+).

**Table 1:** Participant characteristics.

**Donor**	***N***	**Study**	**Experiment**	**Age range**	**HCMV serostatus**
Healthy	10	Local volunteers	Assessment of antiviral activity of lysate/peptide stimulated supernatants in VDA Autologous VDA	30–63	6 seropositive 4 seronegative
Healthy	11	ARIA	Assessment of antiviral activity of lysate/peptide stimulated supernatants in VDA	23–76	9 seropositive 2 unknown
Healthy	16	AQUARIA	Autologous VDA	31–76	10 seropositive 6 seronegative
Kidney Tx (D+R–)	7	AMC	FluoroSpot analysis of IFNγ-producing CD3^+^ T cells	21–66	7 seronegative
Kidney Tx (D+R+)	4	Royal Free	Autologous VDA FluoroSpot analysis of IFNγ-producing CD3^+^ T cells	29–54	4 seropositive

### Recruitment: Healthy Volunteers

HCMV seronegative and seropositive individuals were recruited in younger (<40 years of age) and older (65> years of age) groups, donating either PBMC (ARIA study; Jackson et al., 2017b) or PBMC and autologous primary dermal fibroblasts (AQUARIA study).

HCMV seropositive and seronegative donors were recruited in three stages. Ten donors were recruited locally with ethical approval from the Cambridge Central Research Ethics Committee (97/092). Eleven donors were previously recruited by the NIHR BioResource Centre Cambridge through the ARIA study (Jackson et al., 2017b), with ethical approval from the Cambridge Human Biology Research Ethics Committee (HBREC.2014.07). Sixteen donors were recruited by the NIHR BioResource Centre Cambridge through the AQUARIA study, with ethical approval from the North of Scotland Research Ethics Committee 1 (NS/17/0110). Donors were excluded if they were receiving immunosuppressive therapy, e.g., cyclosporins or methotrexate.

In each case, informed written consent was obtained from all volunteers in accordance with the Declaration of Helsinki.

Healthy volunteers are summarized in Table 1.

### Isolation of Human Dermal Fibroblasts

Primary human dermal fibroblasts (HDFs) were obtained from individual donors in the AQUARIA study and the D+R+ kidney transplant cohort. A 2-mm punch biopsy was obtained from each donor. HDFs were grown out from this biopsy following a previously published protocol (Poole et al., 2014), modified to use DMEM (Poole et al., 2020).

### Peripheral Blood Mononuclear Cell (PBMC) Isolation

Peripheral blood mononuclear cells were isolated from heparinized blood samples using Histopaque®-1077 (Sigma-Aldrich, Poole, UK) or Lymphoprep (Axis-shield, Oslo, Norway) density gradient centrifugation. HCMV serostatus was assessed using an IgG enzyme-linked immunosorbent assay (Trinity Biotech Plc, Co., Wicklow, Ireland). Patient, ARIA and local donor PBMC were frozen in liquid nitrogen in a 10% dimethyl sulfoxide (DMSO) (Sigma-Aldrich) and 90% SeraPlus fetal bovine serum (PAN Biotech, Wimborne, UK) solution; AQUARIA PBMC were frozen in a serum-free freezing media composed of 60% IMDM (Iscove's Modified Dulbecco's Medium, Sigma), 10% DMSO, and 30% Panexin serum replacement (PAN Biotech). Frozen PBMC were rapidly thawed, and the freezing medium was diluted into 10 ml of fresh X-Vivo 15 (Lonza, Slough, UK). PBMC were incubated at 37°C with 10 U/ml DNase (Benzonase, Merck-Millipore via Sigma-Aldrich) for 1 h, followed by resuspension in fresh media and an overnight incubation at 37°C.

### Specific Cell Subtype Isolation

PBMC were enriched for CD8^+^ T cells or NK cells by magnetically activated cell sorting (MACS) using CD8^+^ T cell (130-096-495) or NK cell (130-092-657) isolation kits (Miltenyi Biotech, Woking, UK), according to the manufacturer's instructions. Cells were separated by use of an autoMACS Pro separator (Miltenyi Biotech). The efficiency of depletion was determined by staining cells as described in the phenotyping method below. Depletions performed in this manner resulted in 0–0.3% residual CD4^+^ T cell content of CD8^+^ cell fractions, and 0.4–3.7% CD3^+^ T cell contamination of NK cell fractions.

### Fluorescently Labeled Merlin (mCherry-P2A-UL36 [vICA], GFP-UL32 [pp150])

The virus used in this study was based on a BAC cloned version of HCMV strain Merlin (Wilkinson et al., 2015). This contains the complete wildtype HCMV genome, with the exception of point mutations in RL13 and UL128, which enhance growth in fibroblasts (Stanton et al., 2010). Two genes were tagged with fluorescent markers. UL36 was linked to mCherry via a P2A linker. This arrangement results in expression of mCherry at immediate early times (Nightingale et al., 2018). UL32 was linked directly to GFP via a six amino-acid linker, and results in GFP expression at late times (Weekes et al., 2014). Both constructs were generated by recombineering as previously described (Stanton et al., 2008). In both cases, a recombineering cassette expressing kanR, lacZa, and rpsL was first inserted at the site of modification, following PCR amplification using primers in Supplementary Table 3. For GFP fusions to UL32, the primers listed in Supplementary Table 3 were used to amplify GFP and switch the recombineering cassette for the linker-GFP sequence. For insertion of P2A-mCherry after UL36, the insertion was gene synthesized by Geneart, digested to release it from its original vector, gel purified, and used to replace the recombineering cassette. All constructs were sequenced by Sanger sequencing across the site of modification, including any inserted sequence.

### Virus Propagation

HCMV strain Merlin mCherry-P2A-UL36 GFP-UL32 was grown in human foreskin fibroblasts (HFF), following the protocol in Wills et al. (2005). The infectious titer and PFU were calculated using the method in Jackson et al. (2017a).

### HCMV-Infected Cell Lysate and Control Fibroblast Lysate

One T175 flask of MRC-5 fibroblasts was maintained in DMEM [Sigma, Poole, UK] with 10% FCS [PAA, Linz, Austria] and pen-strep [10^5^ IU penicillin/L, and 100 mg streptomycin/L (Invitrogen Life Technologies)]. When cells were 80% confluent, they were infected with dual-color Merlin and the virus was propagated for 14 days at 37°C in a 5% CO_2_ humified atmosphere. When 80% of cells were mCherry+ or mCherry+GFP+ by fluorescent microscopy, the infected cells were harvested using trypsin. A second T175 flask of MRC-5 fibroblasts was used as control fibroblast lysate and was harvested when cells were 95% confluent. Cells were resuspended in 10 ml DMEM with 10% FBS and placed in a 50 ml Falcon. Cells were subjected to three freeze-thaw cycles to lyse the cells, alternating between a dry-ice/ethanol bath and 37°C water bath. The lysed cell suspension was then centrifuged for 7 min at 700RPM and the supernatant (the lysate) drawn off and frozen in 1 ml aliquots were stored at −80°C until further use. Lysates were heat-treated at 56°C for 30 min before subsequent application (Nokta et al., 1996; Hodinka, 2007).

### HCMV ORF Peptide Pools

Seven HCMV proteins were selected based on previously demonstrated immunogenicity (Jackson et al., 2014, 2017a) and peptide libraries comprising consecutive 15mer peptides overlapping by 10 amino acid were synthesized by ProImmune PEPScreen (Oxford, UK) and JPT Peptide Technologies GmbH (Berlin, Germany). Individual lyophilized peptides were reconstituted and used as previously described (Jackson et al., 2014) in peptide pools at a concentration of 5 μg/ml/peptide. The peptide pools covered the following ORFs: gB (UL55); pp65 (UL83) and UL144; IE1 (UL123) and IE2 (UL122); pp71 (UL82) and US3.

### Cytokine Quantification by Enzyme-Linked Immunosorbent Assay (ELISA)

The Human IFNγ ELISA MAX Standard Set (Biolegend, London, UK) was used to quantify IFNγ concentrations in supernatants. ELISAs were performed according to the manufacturer's recommended protocol.

### Detection of Cytokine Production by FluoroSpot

To maximize available cell numbers, 1 × 10^5^ total PBMC were suspended in X-VIVO 15 (Lonza, Slough, UK) supplemented with 5% Human AB serum (Sigma Aldrich). PBMC were incubated in pre-coated human IFNγ and IL-10, or IFNγ, IL-10, and TNFα FluoroSpot plates (Mabtech AB, Nacka Strand, Sweden) in triplicate with ORF peptide pools (final peptide concentration 2 μg/ml following dilution with X-Vivo-15) and an unstimulated and positive control mix [containing anti-CD3 and anti-CD28 (Mabtech AB) or colloidal anti-CD3 and anti-CD28 (Human T cell TransAct, Miltenyi Biotech), at 37°C for 48 h]. Cells and media were decanted from the plate and the assay developed following the manufacturer's protocol. Developed plates were read using an AID iSpot reader (Oxford Biosystems, Oxford, UK) and counted using AID EliSpot v7 software (Autoimmun Diagnostika GmbH, Strasberg, Germany).

Donor results were discounted from further analysis if there was >1,000 spot forming units (sfu) per well. The sfu response in the positive control (mitogen stimulation) wells had to be at least 100 sfu/well greater than the background sfu/well, otherwise the sample failed quality control and was excluded. The mean sfu of triplicate wells were converted to sfu per 10^6^ cells, the mean background response (sfu/10^6^ cells) was deducted from the mean responding wells. The cut off to define a positive IFNγ response was responses >100 sfu/10^6^ cells, negative responses were below this. The cut off was determined by comparing the distribution of the responses from HCMV seropositive and seronegative donors to HCMV protein stimulation and the positive control in our previous ARIA study results (Jackson et al., 2017b).

### Generation of Supernatants Following Lysate Stimulation

3 × 10^5^ total PBMC were suspended in X-VIVO 15 (Lonza, Slough, UK) in a 5 ml polypropylene tube and stimulated with heat inactivated HCMV [Merlin]-infected fibroblast lysate or uninfected fibroblast lysate, and positive control mixes (anti-CD3 and anti-CD28, as above). They were incubated at 37°C, in a 5% CO_2_ atmosphere for 48 h. Tubes were spun for 10 min at 2000RPM to pellet the PBMC. Two milliliters media (the supernatant) was then removed from each tube without dislodging the cell pellet and frozen at −80°C.

### Generation of Supernatants Following Peptide Stimulation

3 × 10^5^ total PBMC were suspended in X-VIVO 15 (Lonza, Slough, UK) in a 5 ml polypropylene tube and stimulated with ORF peptide pools and unstimulated (X-VIVO 15) and positive control mixes. They were incubated at 37°C in a 5% CO_2_ atmosphere for 48 h and supernatants harvested as above.

### Virus Dissemination Assay: Non-autologous

Human fetal foreskin fibroblasts (HFFFs) were maintained in DMEM [Sigma, Poole, UK] with 10% FCS [PAA, Linz, Austria] and pen-strep [10^5^ IU penicillin/L, and 100 mg streptomycin/L (Invitrogen Life Technologies)]. HFFFs were seeded at 2.5 × 10^4^ cells/well in a 96-well plate. After 48 h, cells were confluent and were infected at a low MOI (0.01) with mCherry-GFP-Merlin. After a further 24 h, 75 μl of lysate or peptide-stimulated supernatant was added to each well. After 9–11 days, cells were harvested with trypsin and fixed in a 2% PFA solution for flow cytometry analysis of viral dissemination.

Viral spread in each well was determined as a percentage of control wells lacking supernatants using the following equation ([Experimental % of infected cells – background % of HFFF-only control]/[% of infected HFFF control without effector cells/supernatants – background % of HFFF-only control]) × 100.

### Virus Dissemination Assay: Autologous

Human primary dermal fibroblasts (HDFs) were maintained in DMEM [Sigma, Poole, UK] with 20% FCS [PAA, Linz, Austria] and pen-strep [10^5^ IU penicillin/L, and 100 mg streptomycin/L (Invitrogen Life Technologies)]. Where paired HDFs and effector cells (PBMC; CD8^+^; NK) were available from healthy volunteers, 1 × 10^4^ HDFs were seeded in each well of a half-area 96-well plate (Greiner Bio-One, Stroudwater, UK). After 48 h, cells were confluent and were infected at a low MOI (0.01) with mCherry-GFP-Merlin HCMV. After a further 24 h, cells were seeded at a range of effector to target ratios in X-Vivo 15 (Lonza, Slough, UK). After 10–14 days, PBMC or effector cells were washed off and HDFs were harvested with trypsin and fixed in a 2% PFA solution for flow cytometry analysis of viral spread.

Viral spread in each well was determined as described previously.

### Phenotyping

10^5^ total PBMC was stained in separate tubes with two phenotyping cocktails containing 2 μl of each antibody:

T cell antibody mix: anti-CD3—fluorescein isothiocyanate (FITC), clone UCHT1; anti-CD4—phycoerythrin (PE), clone RPA-T4; anti-CD8a-peridinin-chlorophyll protein—cyanine 5.5 (PerCP Cy5.5), clone RPA-8a (all BioLegend, London, UK), LIVE/DEAD Fixable Far Red Dead Cell Stain Kit (Thermo Fisher Scientific).

NK cell antibody mix: anti-CD3-FITC; anti-CD8-PerCP Cy5.5 (both as before); anti-CD56-PE, clone B159 (BD Pharmingen); LIVE/DEAD Fixable Far Red Dead Cell Stain Kit.

### Flow Cytometry

Flow cytometry analysis of dual-color virus dissemination was performed on the BD Fortessa2 or Thermo Fisher Attune NxT flow cytometers. PBMC phenotyping was performed on the BD Accuri C6 flow cytometer. Data were analyzed with FlowJo v10 (Becton Dickinson, Wokingham, UK).

### Statistical and Graphical Analysis

Data presentation and statistical analyses was performed using GraphPad Prism v8. Statistical significance for one-tailed *T*-tests was determined with an alpha = 0.05, without assuming a consistent standard deviation between populations.

## Results

### Longitudinal Detection of HCMV-Specific IFNγ CD3^+^ T Cell Responses in D+R– Kidney Transplant Recipients Did Not Predict Resolution of Viraemia

**Figure 1:**
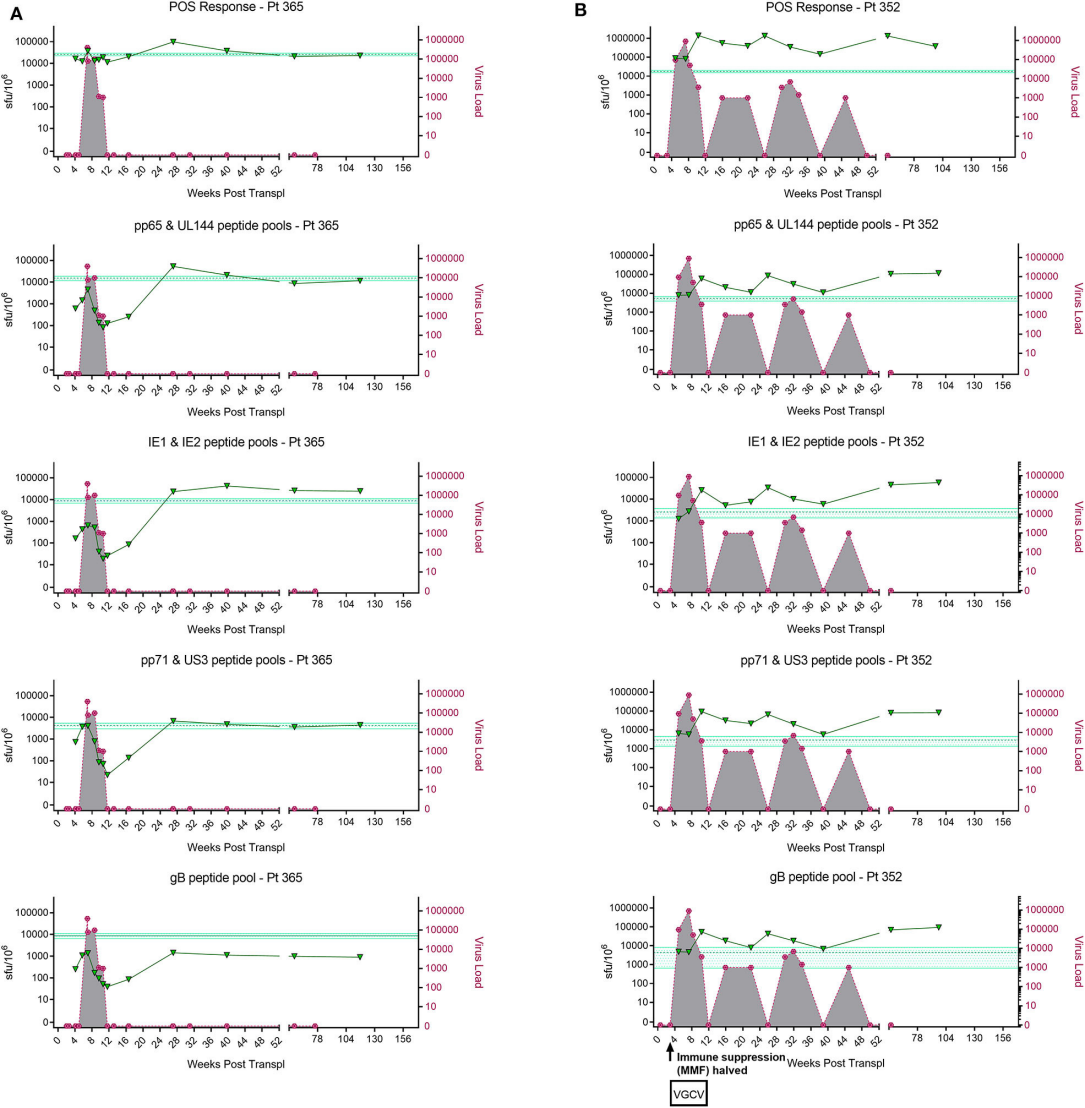
Analysis of longitudinal HCMV virus load and HCMV specific CD3+ T cell IFNγ responses of D+R– Kidney transplant patients. Two example D+R– kidney transplant patients with primary HCMV infection, T cell responses (spot forming units per 10^6^ CD3^+^ T cells) were measured by IFNγ FluoroSpot (green triangles connected by a solid line) to HCMV peptide pools covering pp65 and UL144, IE1 and IE2, pp71 and US3, and gB, as well as a polyclonal T cell stimulation as a positive control (“POS”). Virus load (copies/ml blood) was measured by QNAT of HCMV DNA (pink hexagons connected by a dashed line). Cyan lines show the mean magnitude of response (±standard error) of CD3+ T cell IFNγ responses seen in healthy seropositive individuals in the same age decade as the transplant recipient for each peptide pool (Jackson et al., 2017b). **(A)** Patient 365 is an example of a D+R– patient with resolution of DNAemia following the emergence of detectable CD3^+^ IFNγ responses to four HCMV lytic peptide pools. **(B)** Patient 352, in contrast, had DNAemia which recurred several times, despite also developing detectable HCMV specific CD3^+^ T cell IFNγ responses which are comparable in frequency to those seen age-matched in healthy seropositives.

IFNγ T cell responses to HCMV *in vitro* have previously been used as a surrogate measure of the level of cell-mediated immunity to HCMV *in vivo*. To begin testing this assumption, we characterized the development of IFNγ T cell responses following primary HCMV infection. To do this, CD3^+^ T cell IFNγ responses to HCMV peptide pools were measured by Fluorospot on a cohort of D+R– kidney transplant patients, all of whom experienced primary HCMV infection following transplantation. In the seven patients studied, all developed robust IFNγ responses to a range of HCMV peptide pools, covering lytic proteins gB, IE1, IE2, US3, pp65, UL144, and pp71, responses enumerated by FluoroSpot (Figure 1 and Supplementary Figure 1).

However, these longitudinal analyses revealed examples of two broadly distinct patterns of response in this cohort (Figure 1). In some patients (3/7 e.g., Pt365) (Figure 1A), we observed the generation of a HCMV-specific IFNy CD3^+^ T cell response which was sustained over time and correlated with the resolution of initial viraemia. Subsequent episodes of viraemia where not observed after this response became detectable. In other patients, viraemia recurred once (3/7 e.g., Pt574; Supplementary Figure 1) or several times (1/7 e.g., Pt352; Figure 1B), despite detectable HCMV-specific IFNy CD3^+^ T cell responses of similar magnitude being present at the times of recrudescence. As such, the results show that in this group of patients, CD3^+^ T cell IFNγ responses as measured by FluoroSpot were not necessarily predictive of the resolution of CMV viraemia, even when a broad selection of highly immunogenic HCMV peptide pools was used. Importantly, a comparison of the magnitude of these responses with a previously studied cohort of healthy seropositive donors in the same age range as each transplant recipient (Jackson et al., 2017b) revealed the CD3^+^ T cell IFNγ FluoroSpot responses of these seven D+R– patients were of a similar magnitude and breadth arguing that these patients can generate substantial T cell responses when measured by IFNy Fluorospot.

### Determination of Antiviral Efficacy of HCMV Antigen Stimulated PBMC and Its Correlation to IFNγ Detection-Based Assays

We speculated that one explanation for the inconsistent predictive power of simply measuring CD3^+^ HCMV-specific T cell IFNγ responses as a correlate with *in vivo* HCMV control could be that other cytokines and secreted factors may also play an important role in antiviral T cell immunity to HCMV (Nachtwey and Spencer, 2008; Mason et al., 2013; Siewiera et al., 2013).

**Figure 2:**
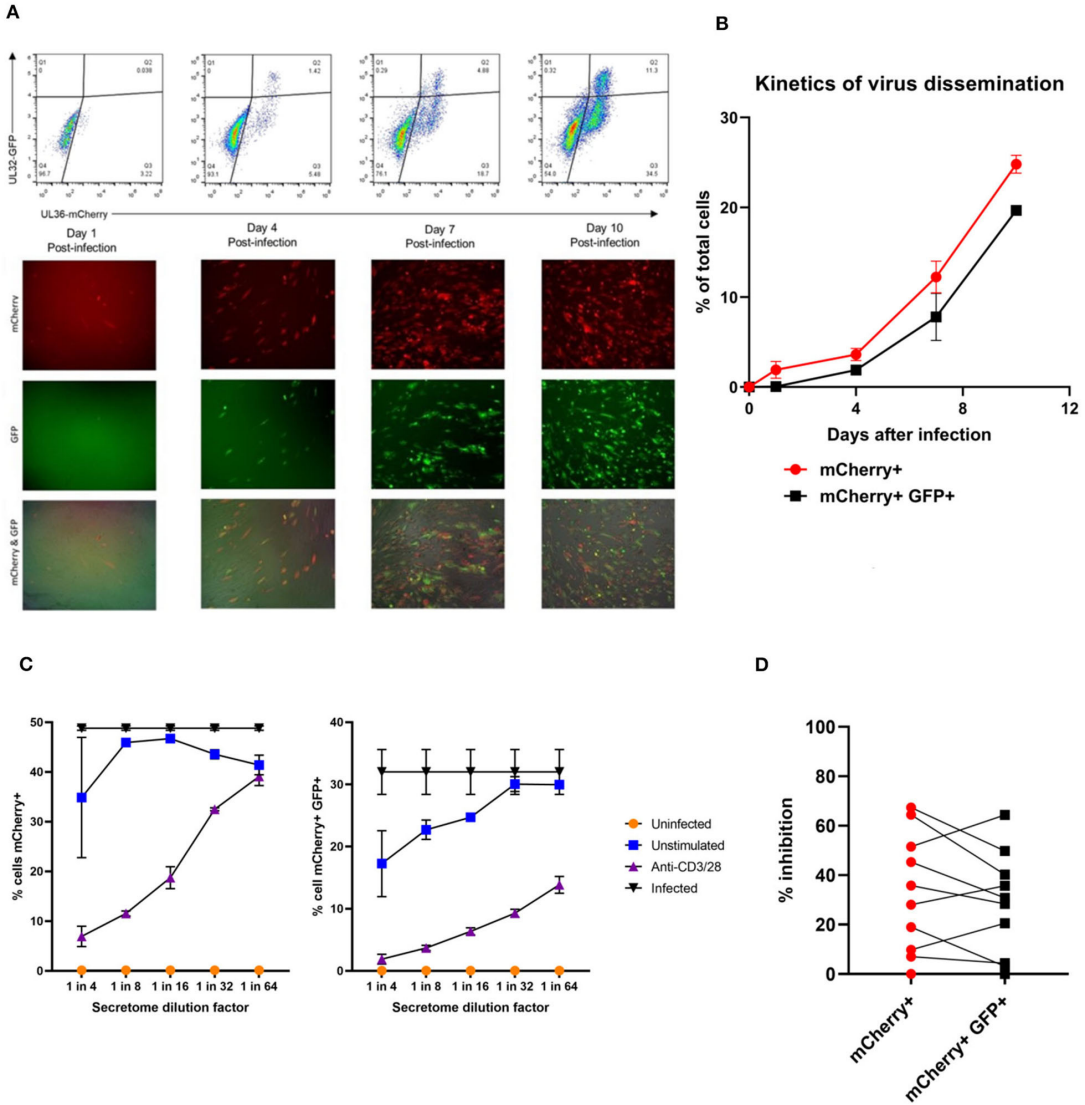
Quantification of HCMV dissemination and antiviral effect of polyclonally stimulated T cells. **(A)** Viral dissemination assay using a dual-fluorescently tagged HCMV strain (Merlin mCherry-P2A-UL36 [vICA], GFP-UL32 [pp150]). Following a low MOI (0.01) infection of indicator fibroblasts, infected cells become mCherry+ as the virus enters the immediate-early life cycle, and later become mCherry+ GFP+ as the virus enters the late life cycle. The percentage of cells infected can be visualized by fluorescent microscopy and quantified by two color flow cytometry. **(B)** Kinetics of virus dissemination in HFFF cells. Virus dissemination has been quantified by flow cytometry at various time points post-infection based on mCherry+ GFP– cells and on mCherry+ GFP+ cells. **(C)** Analysis of antiviral activity of supernatant from anti-CD3/CD28 stimulated T cells in PBMC (supernatant donor ARIA060). Fibroblasts were infected at 0.01 MOI and co-cultured with dilutions of supernatant, following 10 days of incubation fibroblasts were harvested and analyzed for mCherry and GFP expression by flow cytometry. **(D)** Analysis of antiviral activity of supernatant from anti-CD3/CD28 stimulated T cells in PBMC from 10 independent donors, diluted 1:4. Fibroblasts were infected at 0.01 MOI and co-cultured with dilutions of supernatant, following 9–11 days of incubation fibroblasts were harvested and analyzed for mCherry and GFP expression by flow cytometry.

In order to address this the capacity of the CD3^+^ T cells to control viral replication we adapted a viral dissemination assay (VDA) previously used by our group to quantify the spread of virus through indicator fibroblasts co-cultured with NK cells or CD8^+^ T cells (Jackson et al., 2014; Chen et al., 2016). Using a dual mCherry-GFP expressing strain of HCMV (Merlin), the progression of infection can be assayed. Infected cells express mCherry from immediate-early times post-infection (linked to UL36-P2A-mCherry) and are both mCherry and GFP+ (fused to UL32 [pp150 – tegument protein]) at late times post-infection. Following a low MOI infection, the virus spreads through fibroblasts over time. The infected mCherry+ and mCherry+ GFP+ cells can be visualized using fluorescent microscopy and enumerated by two color flow cytometry (Figure 2A), quantifying viral dissemination kinetics over a series of time points (Figure 2B).

Prior to the specific analysis of the supernatants derived from T cells stimulated by HCMV antigens, we first determined whether supernatant from PBMC incubated with anti-CD3 and anti-CD28 antibodies to polyclonally activate the T cells (Trickett and Kwan, 2003) was antiviral compared to unstimulated PBMC control from the same donor. To test for any antiviral activity, supernatants were added to cultures of HFF fibroblasts that had been previously infected with HCMV overnight at low MOI (0.01). The data show that supernatants taken from antibody-stimulated T cells reduced the percentage of both mCherry+ fibroblasts and mCherry+ GFP+ fibroblasts in a titratable manner (Figure 2C). Although some antiviral activity was evident using supernatants from unstimulated PBMC (particularly when fibroblasts were analyzed for GFP expression), this activity was considerably lower than following polyclonal T cell activation. An analysis of supernatants from activated T cells from multiple donors (*n* = 10) revealed that the level of antiviral activity induced varied considerably between donors (Figure 2D).

It was noted that following overnight infection, and addition of PBMC derived supernatants, that the initial infected cells (identified as mCherry+) proceeded to late gene expression (i.e., became GFP+) after a further 48 h incubation, even in the presence of a supernatant identified as antiviral. We hypothesized, therefore, that the antiviral effects observed were due to inhibition of the secondary infections that occur in our VDA rather than an inhibition of the first round of infected cells. These subsequent rounds of infection (spread) can be measured if immediate early gene expression occurs (mCherry+ cells) and/or subsequent progression to late viral gene expression by GFP expression is impacted. As such, two scenarios are possible: uninfected cells incubated with these supernatants might become refractory to HCMV infection or induced into an antiviral state which disrupts normal temporal viral gene expression between immediate early and late viral gene expression. We therefore subsequently define a reduction in the relative percentage of cells which are mCherry+GFP– as a reduction in virus spread in the culture. If a reduction in the relative percentage of cells which are mCherry+ and GFP+ is observed (and thus a reduction in progression to late gene expression), we can use this as a proxy for a reduction in the number of new infections in the VDA capable of producing new infectious virus particles.

**Figure 3:**
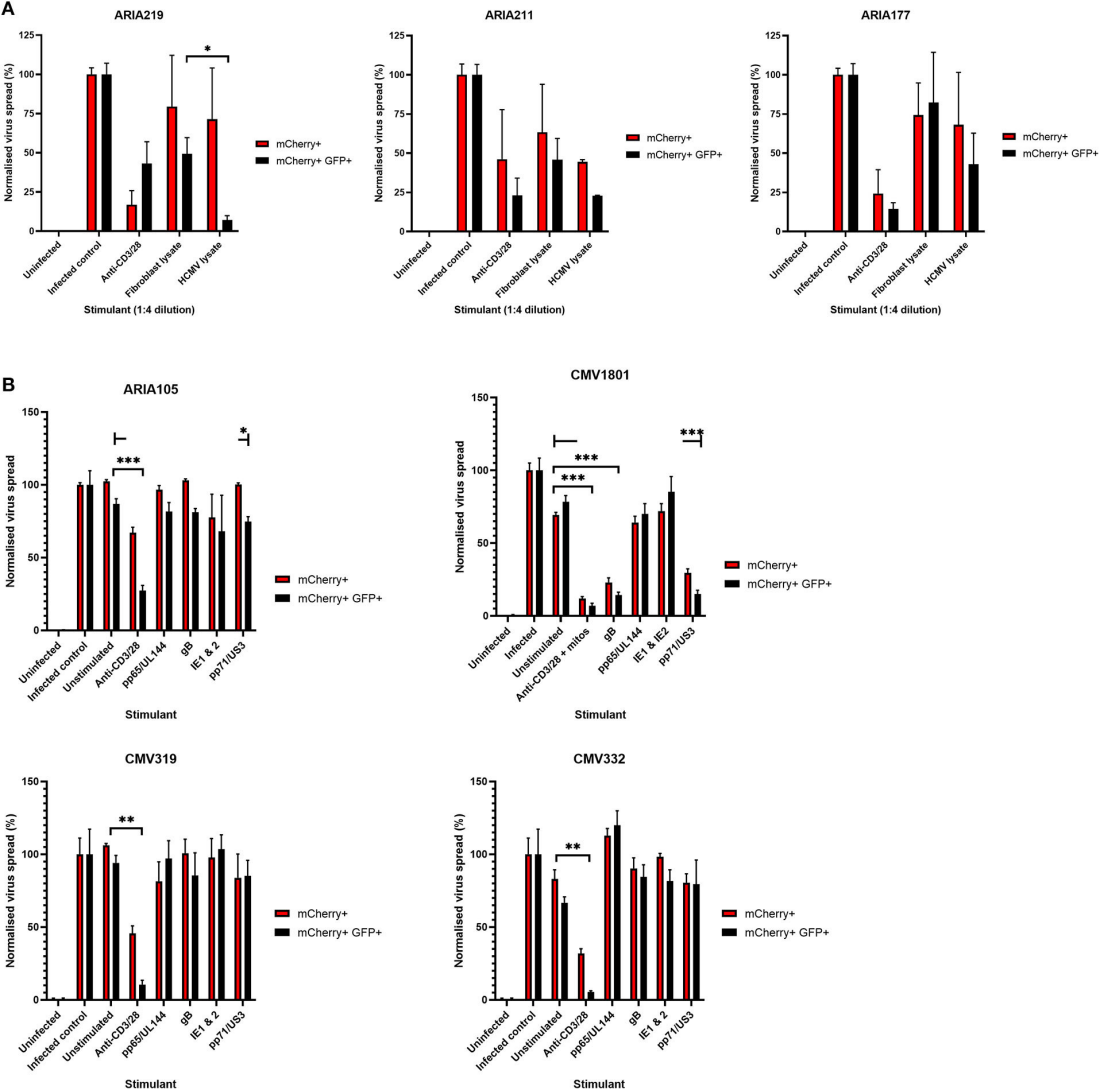
Analysis of the antiviral activity of PBMC stimulated with HCMV-infected fibroblast lysates and pools of HCMV synthetic peptides specific for pp65, UL144,Gb, IE1,IE2, pp71, and US3. **(A)** Antiviral activity of supernatants derived from PBMC from three different donors stimulated with HCMV infected or uninfected fibroblast lysates. PBMC were also stimulated with anti-CD3/CD28 antibodies to generate a positive control antiviral supernatant. Fibroblasts were infected at 0.01 MOI and co-cultured with 1:4 dilution of supernatant, following 9–11 days of incubation fibroblasts were harvested and analyzed for mCherry and GFP expression by flow cytometry. Significance determined was by one-tailed *T*-test, *p* < 0.05. **(B)** Antiviral activity of HCMV peptide pools covering pp65 and UL144, IE1 and IE2, pp71 and US3, and gB, as well as a polyclonal anti-CD3/CD28 antibody T cell stimulation as a positive control on five independent HCMV seropositive donors. Significance determined by one-tailed *T*-test. Key: *p < 0.05;**p < 0.005;***p < 0.0005.

Having established that we could generate and define antiviral supernatants by polyclonal T cell stimulation of PBMC, we then wished to study the HCMV-specific secreted antiviral immune response of PBMC from healthy seropositive volunteers. Healthy donor PBMC was stimulated with heat-treated HCMV-infected fibroblast lysate (“lysate”), and harvested supernatants tested for antiviral activity in our VDA. The results show that the outcome of PBMC stimulation is variable. Some seropositive donors (e.g., ARIA219) generated HCMV-specific antiviral supernatants, as they reduced the number of mCherry+GFP+ fibroblasts. In contrast, supernatants from PBMC taken from donor ARIA177 show comparable inhibition of virus by both the uninfected control fibroblast lysate and the HCMV-infected lysate stimulation. Other donors had a weak antiviral response to HCMV, such as ARIA211 (Figure 3A). ARIA177 supported the hypothesis that non-self-antigens from the fibroblasts used to produce the lysate may have stimulated a non-specific antiviral response from some donors—which would be consistent with our observations with supernatants from polyclonally stimulated T cells. We therefore adapted this assay to use the gB, IE1, IE2, US3, pp65, UL144, and pp71 pools of immunodominant HCMV peptides, recognized by both CD8^+^ and CD4^+^ T cells for PBMC stimulation (Sylwester et al., 2005; Jackson et al., 2017b).

**Figure 4:**
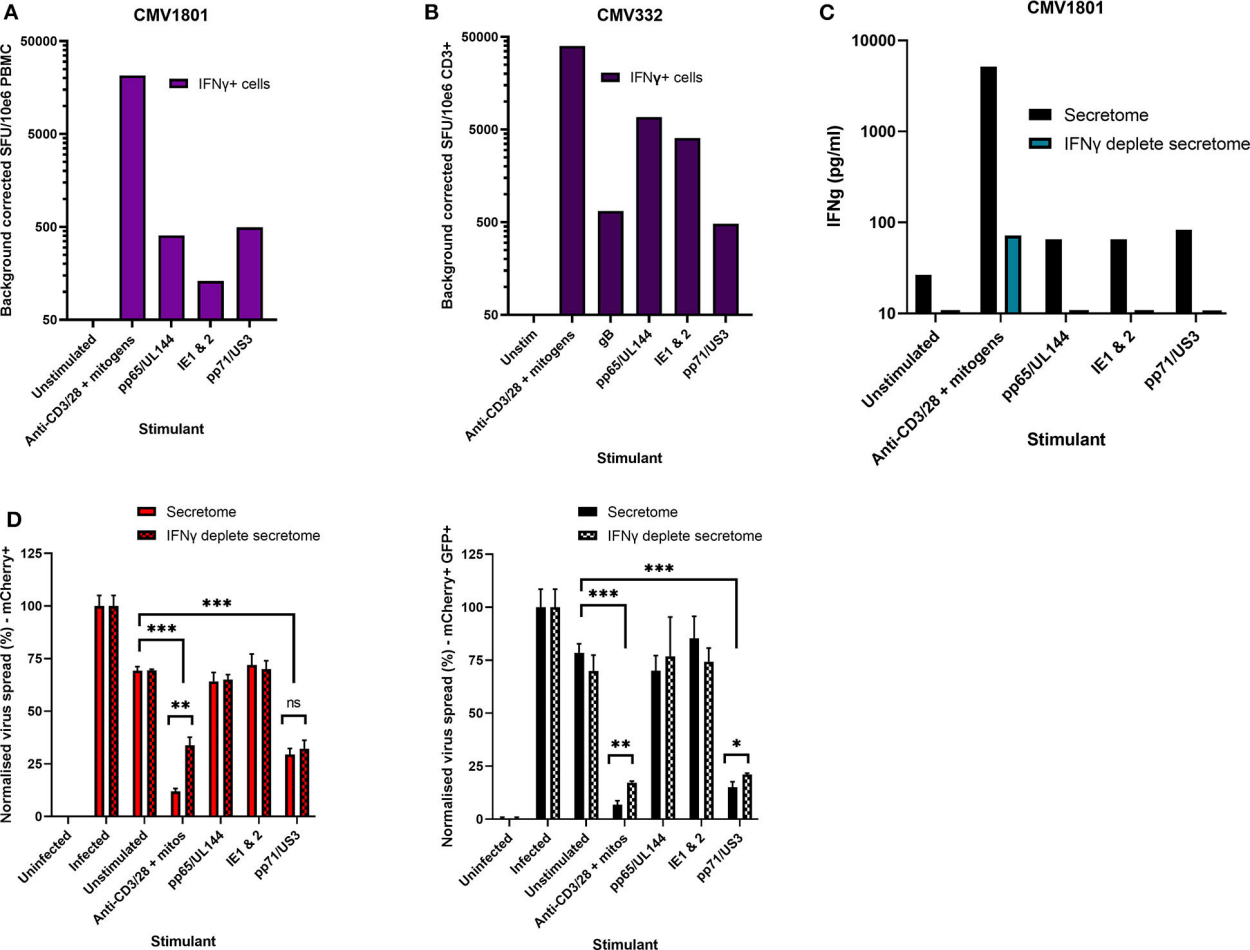
Analysis of HCMV specific IFNγ FluoroSpot responses and antiviral activity of HCMV peptide stimulated supernatants with and without IFNγ depletion. **(A)** IFNγ FluoroSpot responses to HCMV peptide pools covering pp65 and UL144, IE1 and IE2, pp71 and US3, and gB, as well as a polyclonal anti-CD3/CD28 antibody T cell stimulation as a positive control of PBMC from donor CMV1801, calculated as spot-forming units (SFU) per 10^6^ PBMC (background corrected). **(B)** IFNγ FluoroSpot responses to HCMV peptide pools covering pp65 and UL144, IE1 and IE2, pp71 and US3, and gB, as well as a polyclonal anti-CD3/CD28 antibody T cell stimulation as a positive control of PBMC from donor CMV332, calculated as spot-forming units (SFU) per 10^6^ PBMC (background corrected). **(C)** The IFNγ concentration of supernatants following peptide stimulation (black) or after IFNγ depletion by anti-IFNγ-coated FluoroSpot (cyan), measured by ELISA. **(D)** The effect of IFNγ depletion on the antiviral activity of PBMC from donor CMV1801 stimulated with HCMV peptide pools for pp65/UL144, IE1&2, and pp71/US3 or anti-CD3/CD28 antibody. Bars labeled “IFNγ deplete” were harvested from anti-IFNγ antibody-coated FluoroSpot plates and added to a VDA in parallel with supernatants generated with the same stimulants and PBMC cell number. Significance determined by one-tailed *T*-test. Key: *p < 0.05;**p < 0.005;***p < 0.0005; ns, Not significant.

Healthy seropositive donor PBMC was thus stimulated with these HCMV peptide pools for 48 h and the supernatant harvested and added to the VDA at 24 hpi. Here supernatants from unstimulated PBMC were used as a negative control and polyclonal stimulation was used as a positive control for antiviral activity. The results show that two of the four donors tested produced HCMV-specific antiviral supernatants in response to stimulation with one or more of the peptide pools, while two other donors (CMV319 and CMV332) did not produce an antiviral response following peptide stimulation of PBMC (Figure 3B). Hypothetically, the supernatants harvested from donors CMV319 and CMV332 may not have been antiviral in a VDA because these donors did not have memory T cells to any of the HCMV peptides used to stimulate the T cells. To investigate this, we quantified the HCMV peptide specific IFNγ CD3^+^ T cell responses FluoroSpot of PBMC from CMV319 and CMV332, alongside CMV1801. The results show that CMV1801 had positive FluoroSpot responses to pools containing peptides covering pp71/US3 (Figure 4A), CMV332 had positive FluoroSpot responses to pools containing peptides covering pp65/UL144 and IE1&2 (Figure 4B) and CMV319 had positive IFNγ FluoroSpot responses to peptides from IE2 and pp65 (data not shown).

This approach revealed a disconnect between the detection of HCMV specific IFNγ responses (by FluoroSpot) and the ability of these supernatants to exert antiviral activity. Both CMV1801 and CMV332 had positive IFNγ CD3^+^ T cell responses to multiple HCMV peptide pools and in some cases at higher frequency (e.g., pp65/UL144 and IE1/2), yet the supernatant from CMV332 was not antiviral in the VDA (Figures 3B, 4A,B). We therefore speculated that IFNγ was not the key cytokine that determines antiviral activity in the supernatants. To address this, HCMV peptide pools were used to stimulate PBMC from donor CMV1801 for analysis in parallel. Specifically, one in an IFNγ FluoroSpot plate (this would enumerate the T cell response and deplete free IFNγ) and one in a non-antibody coated microtitre plate so that IFNγ was present in the supernatant. Depletion of IFNγ was verified by ELISA (Figure 4C) and also analyzed for antiviral activity by VDA (Figure 4D).

The results show that despite CMV1801 having IFNγ-producing T cells specific for pp65/UL144 and IE1&2 as well as pp71/US3, only supernatants from pp71/US3-stimulated T cells exhibited antiviral activity. Furthermore, this activity remained following depletion of IFNγ from those supernatants. Taken together, these data support our hypothesis that other factors, beyond IFNγ, are important to the secreted antiviral immune response to HCMV. Additionally, their identification may reveal biomarkers of HCMV immunity.

### PBMC and CD8^+^ T Cells Control HCMV in an Autologous *in vitro* Co-culture Viral Dissemination Assay

Thus, far our studies have focused on the anti-viral activity of cytokines produced by stimulated T cells. However, direct T cell-mediated cytotoxicity is an important mechanism of control of viral infection. We previously developed an assay to quantify CD8^+^ T cell and NK cell-mediated antiviral immunity, measuring the lymphocyte-mediated inhibition of dissemination of HCMV through a permissive autologous primary human fibroblast monolayer (Jackson et al., 2014, 2019; Chen et al., 2016). Similar assays used in other laboratories have utilized partially HLA-matched fibroblasts and cytotoxic T lymphocytes (Sinzger et al., 2007) or measured lysis of HCMV-infected autologous fibroblasts with various NK cell clones derived from independent donors (Carr et al., 2002). We therefore wanted to assess the ability of whole PBMC, CD8^+^ T cells, and NK cells from a cohort of seropositive and seronegative donors to control HCMV in a fully autologous experimental system, and to assess the relative contributions of these cell subsets (and their secreted antiviral factors) to that control. This approach also allows us to directly compare the antiviral efficacy of different cell subsets from the same donor in the same experiment.

Primary autologous dermal fibroblasts were infected at a low MOI (0.01), 24 h later these were co-cultured with either total PBMC or purified CD8^+^ T cells or NK cells, across a range of effector to target (E:T) cell ratios. After incubation for 10–14 days, fibroblasts were analyzed by flow cytometry for mCherry and GFP expression. This comparison enabled us to determine the HCMV specificity of this approach when purified CD8^+^ T cells were used from both HCMV seropositive and seronegative donors. The use of whole PBMC enables an assessment of the antiviral capacity of innate immune cells (NK cells and monocytes) as well as adaptive immune cells [CD8^+^ T cells and gamma-delta (γδ) T cells].

**Figure 5:**
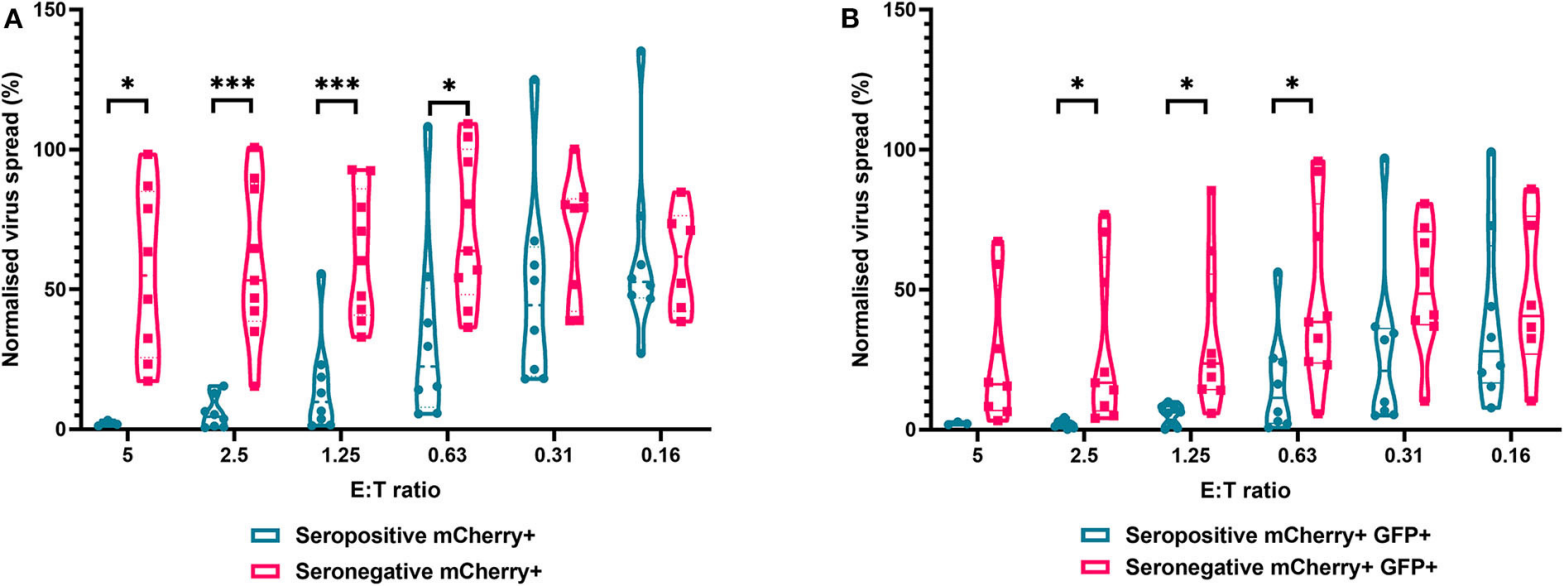
Antiviral activity of whole PBMC from HCMV seropositive and seronegative donors co-cultured with HCMV infected autologous fibroblasts. Violin plots showing results from viral dissemination assays of PBMC co-cultured with HCMV infected fibroblasts for 10–14 days over a range of E:T ratios, fibroblasts were harvested and analyzed for mCherry and GFP expression by flow cytometry. **(A)** Virus spread determined by mCherry+ fibroblasts and **(B)** by mCherry+ GFP+ fibroblasts. Cyan points show the range of control at each E:T for seropositive donors; magenta points are seronegative donors. Significance was determined by one-tailed *T*-test. Key: *p < 0.05;**p < 0.005;***p < 0.0005.

The results are depicted using violin plots which show both the frequency distribution of the VDA by population (e.g., seropositive donor PBMC control of virus spread), and the individual data points for each donor. All data are normalized such that the infected controls for each donor were set as 100% infection, and the uninfected controls were set at 0% infection. At an E:T ratio of 2.5:1, HDFs co-cultured with PBMC from seropositive donors inhibited virus infected cells to between 0.6 and 15.4% of the normalized viral spread (as evidenced by mCherry+ cells) of the infection control. At the same E:T ratio, HDFs co-cultured with PBMC from seronegative donors inhibited virus infected cells to between 34.9 and 100% of the normalized viral spread (mCherry+ cells) of the infection control. The data show PBMC from HCMV seropositive donors (*n* = 8) were significantly better at controlling viral spread (mCherry+ cells) than PBMC from the HCMV seronegative cohort (*n* = 8) at E:T ratios of 5–0.63:1. Similarly, the number of cells that progressed to late stage infection (GFP+) was significantly decreased in the HCMV seropositives vs. the HCMV seronegative cohort at E:T ratios of 2.5–0.63:1 (Figure 5).

**Figure 6:**
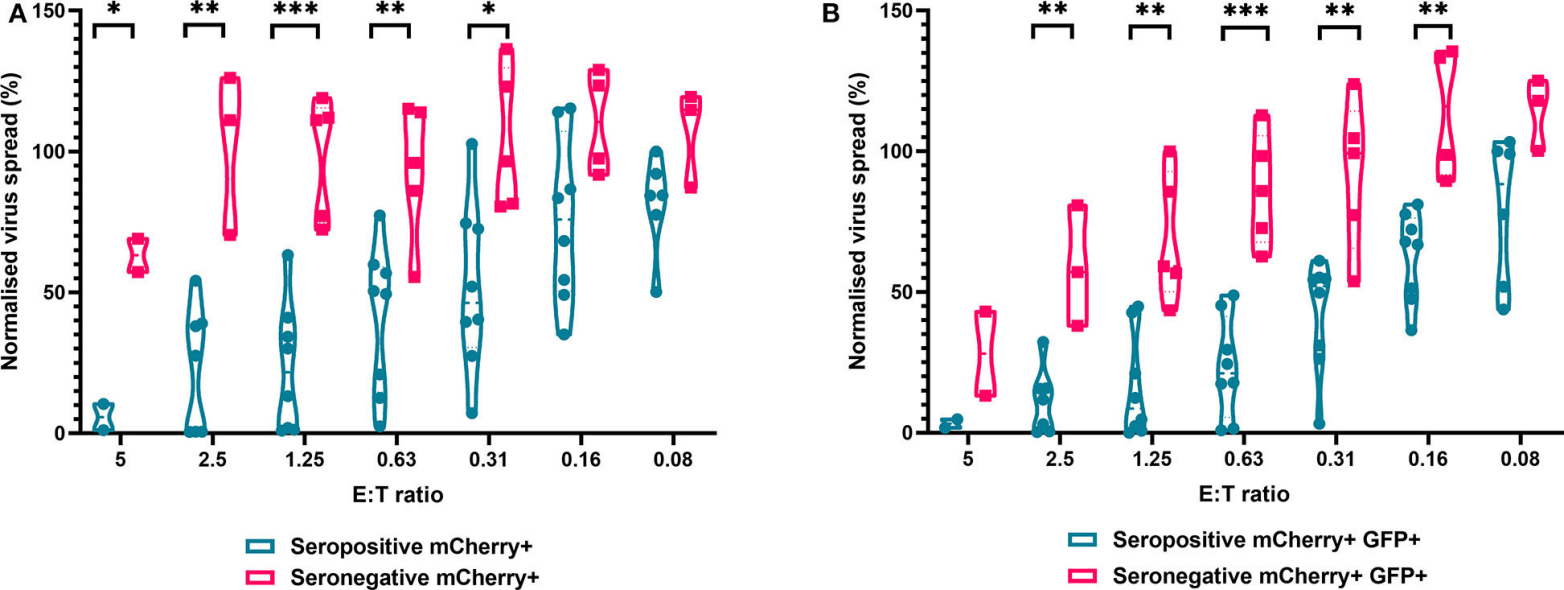
Antiviral activity of purified CD8+ T cells from HCMV seropositive and seronegative donors co-cultured with HCMV infected autologous fibroblasts. Violin plots showing results from Viral dissemination assays of PBMC co-cultured with HCMV infected fibroblasts for 10–14 days over a range of E:T ratios, fibroblasts were harvested and analyzed for mCherry and GFP expression by flow cytometry. **(A)** Virus spread determined by mCherry+ fibroblasts and **(B)** by mCherry+ GFP+ fibroblasts. Cyan points show the range of control at each E:T for seropositive donors; magenta points are seronegative donors. Significance was determined by one-tailed *T*-test. Key: *p < 0.05;**p < 0.005;***p < 0.0005.

We next analyzed the antiviral capacity and specificity of purified CD8^+^ T cells from both HCMV seropositive and seronegative individuals (Figure 6). The data show that CD8^+^ T cells from HCMV seropositive donors were significantly more effective over a range of E:T ratios at inhibiting viral spread (mCherry+) and infectious virus production (GFP+) compared to CD8^+^ T cells from HCMV seronegative donors. We also noted that at the higher E:T ratios CD8^+^ T cells from HCMV seronegative donors were capable of exerting a level of control, but this was lost rapidly, with only HCMV-specific responses observed at E:T ratios of 2.5:1 and lower. At an E:T ratio of 2.5:1, HDFs co-cultured with CD8^+^ T cells from seropositive donors inhibited virus infected cells to between 0.5 and 54.1% of the normalized viral spread (mCherry+ cells) of the infection control. At the same E:T ratio, HDFs co-cultured with CD8^+^ T cells from seronegative donors inhibited virus infected cells to between 70 and 125% of the viral spread (mCherry+ cells) of the infection control.

**Figure 7:**
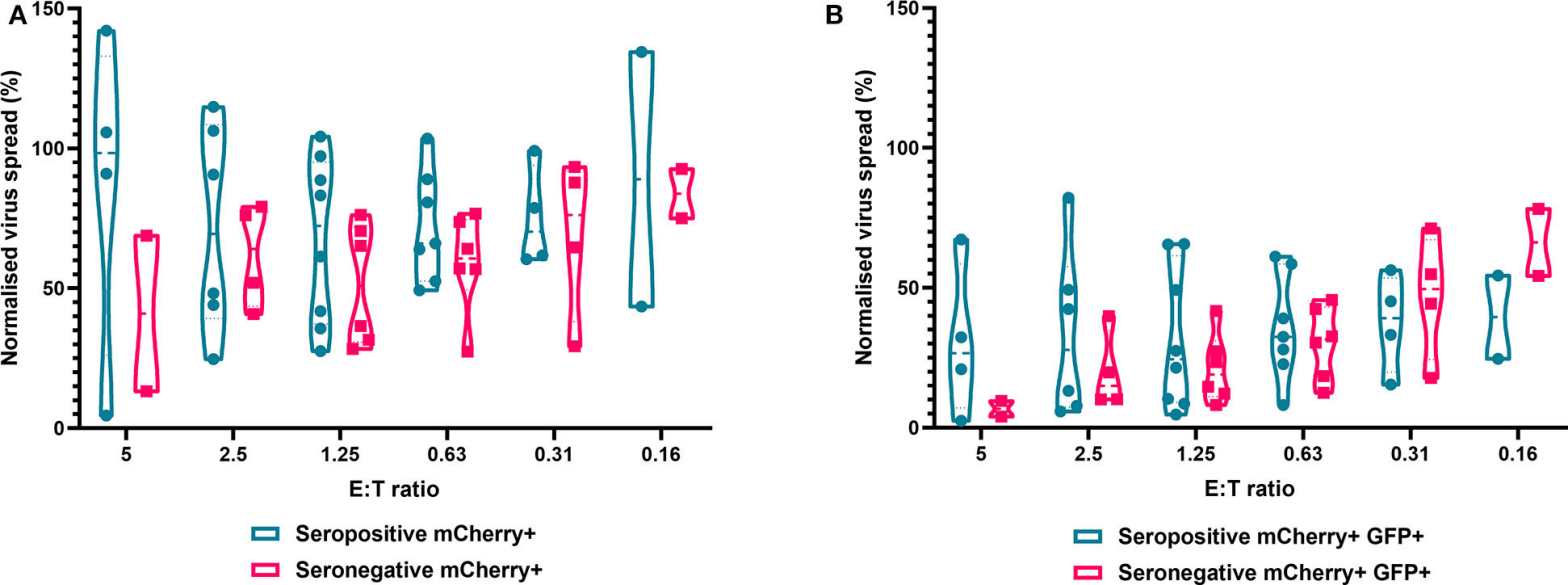
Antiviral activity of purified NK cells from HCMV seropositive and seronegative donors co-cultured with HCMV infected autologous fibroblasts. Violin plots showing results from Viral dissemination assays of PBMC co-cultured with HCMV infected fibroblasts for 10–14 days over a range of E:T ratios, fibroblasts were harvested and analyzed for mCherry and GFP expression by flow cytometry. **(A)** Virus spread determined by mCherry+ fibroblasts and **(B)** by mCherry+ GFP+ fibroblasts. Cyan points show the range of control at each E:T for seropositive donors; magenta points are seronegative donors. Significance was determined by one-tailed *T*-test. Key: *p < 0.05;**p < 0.005;***p < 0.0005.

Finally, we analyzed NK cells purified from PBMC. In contrast to the results observed with PBMC and CD8+ T cells, the ability of NK cells to control HCMV did not vary significantly by donor serostatus (seropositive *n* = 8, seronegative *n* = 5) (Figure 7). It was also notable that NK cell control of virus spread (mCherry+ only cells) was poor compared to the control exerted by CD8^+^ T cells from HCMV seropositive donors over the same range of E:T ratios. At an E:T ratio of 2.5:1, seropositive donors reduced the normalized viral spread (mCherry+ cells) to 24.8–114.8% compared to the infection control, while seronegative donors reduced the normalized viral spread to 40.9–79.2% compared to the infection control. It could also be seen from the violin plots that some donors' NK cells exerted better viral control than others. In order to further illustrate this, we directly compared the data from two HCMV seropositive donors in more detail (Supplementary Figure 2). NK cells from donor AQU002 had high level virus control (controlling virus spread and late gene expression), while NK cells from AQU003 were unable to control virus spread and had significantly less ability to control late virus gene expression.

### HCMV-Seropositive Solid Organ Transplant Recipients (D+R+) Showed Comparable *in vitro* Control of Viral Dissemination to Healthy Controls

In our original analysis of HCMV-specific CD3^+^ T cell IFNγ responses to HCMV lytic peptides, we show that the development of these T cells was not predictive of resolution/recrudescence of viraemia in a number (*n* = 7) of individuals in the D+R– kidney transplant cohort (Figure 1). Approximately half of D+R+ transplant recipients develop viraemia post-kidney or liver transplant, but have shorter duration of viraemia than D+R– patients suggesting pre-existing mature HCMV immune responses are better able to control viraemia (Atabani et al., 2012). In light of our analysis of cell-mediated immunity by IFNγ Fluorospot and VDA in both HCMV seropositive and seronegative healthy individuals, we tested the hypothesis that seropositive transplant recipients who do not develop viraemia have *in vitro* CMI more similar to healthy seropositives than healthy seronegatives in our assays.

To investigate this, we identified four R+ patients who received a D+ organ who were not on antiviral prophylaxis and did not experience detectable viraemia post-transplantation. In this R+ cohort, the HCMV-specific CD3^+^ T cell IFNγ responses as well as total PBMC, CD8^+^ T cells and NK cells by autologous VDA were analyzed. Total PBMC from each R+ individual, collected ~3 months (72–110 days) post-kidney transplant, were stimulated with a panel of HCMV peptide pools as before, and the responses enumerated by IFNγ FluoroSpot (Supplementary Figure 3). The results show all the recipients had a detectable T cell response to at least one of the peptide pools. Recipient R02-00079 made IFNγ T cell responses to all the pools tested, R02-00005 to all but one pool, and R02-00058 and R02-00109 to a single pool each.

**Figure 8:**
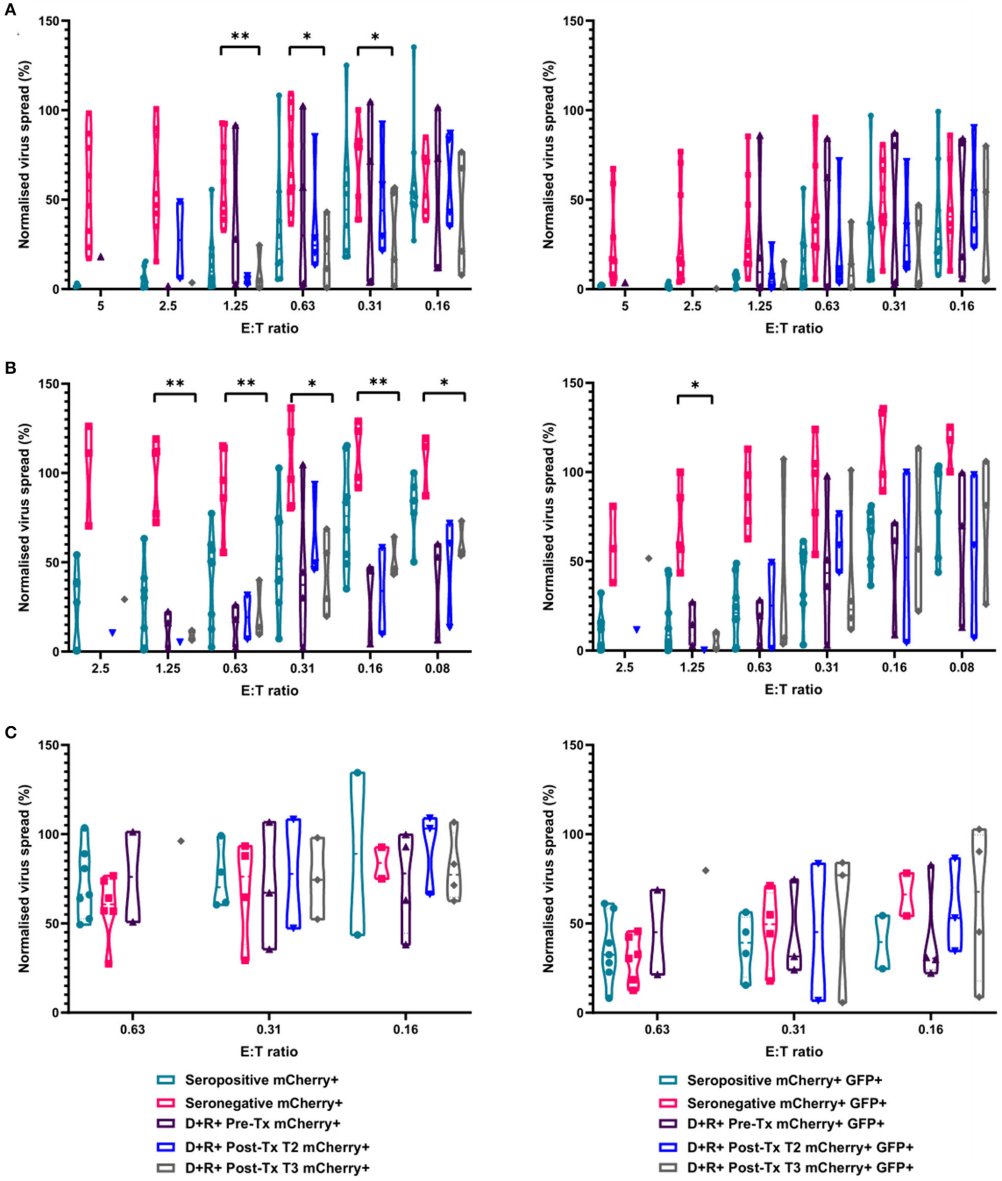
Antiviral activity of PBMC, CD8^+^ T cells, and NK cells from HCMV seropositive and seronegative donors and from non-viraemic D+R+ kidney transplant recipients co-cultured with HCMV infected autologous fibroblasts. Violin plots showing results from Viral dissemination assays of **(A)** PBMC, **(B)** CD8^+^ T cells, and **(C)** NK cells. Cells were co-cultured with HCMV infected fibroblasts for 10–14 days over a range of E:T ratios, fibroblasts were harvested and analyzed for mCherry and GFP expression by flow cytometry. Virus spread determined by mCherry+ fibroblasts and by mCherry+ GFP+ fibroblasts. D+R+ kidney transplant recipients were tested in a VDA from samples collected immediately pre-transplant (T1), at 1–2 months (T2), and 2 months post-transplant. At T3 (post-transplant), PBMC, CD8^+^ T cells, and NK cells were not statistically significantly different from healthy seropositives in their control of virus dissemination. Cyan points show the range of control at each E:T for seropositive donors; magenta points are seronegative donors. Purple points are samples taken immediately pre-transplant. Blue points are samples taken 1–2 months post-transplant. Gray points are samples taken 3 months post-transplant. Significance was determined by one-tailed *T*-test. Key: *p < 0.05;**p < 0.005;***p < 0.0005.

We next determined the anti-HCMV activity of the PBMC, CD8^+^, and NK cells derived from the same R+ kidney transplant recipients. The results demonstrate that PBMC derived from the seropositive kidney transplant recipients 3 months post-transplant were just as effective at controlling HCMV as healthy seropositive PBMC and clearly different from the response in seronegatives (Figure 8A). At an E:T ratio of 1.25, D+R+ PBMC from time point T3 (~3 months post-transplant) controlled the spread of HCMV with a range 0.7–24.5% of the infected control (mCherry+ cells), compared to a range of 1.3–55.6% in healthy seropositives and 33–92.7% in healthy seronegatives. This was also recapitulated in CD8^+^ T cells analyses where again R+ patient cells had a similar pattern of control to CD8^+^ T cells derived from healthy seropositives (Figure 8B). Correspondingly, NK cells from the R+ kidney transplant recipients had a similar range of virus control to both HCMV seropositive and seronegative healthy donors (Figure 8C).

## Discussion

Quantification of the magnitude of the immune response against HCMV has long been investigated as a method for predicting individuals better able to control viral replication in clinical settings. In the context of CMI this has centered on measuring the frequency of IFNy CD3^+^ T cells that recognize HCMV antigens, with the assumption that a direct correlation between frequency and control exists. In this study, we investigated CMI to HCMV in both SOT recipients and healthy controls. These *in vitro* CMI assays suggest that the use of IFNγ as a proxy for HCMV-specific control *in vivo* may not represent the best measure of the antiviral activity of immune cells from immune suppressed SOT recipients. Our view that measurement of IFNγ is not a robust biomarker is supported by two pieces of evidence: firstly, a clear lack of correlation between CD3^+^ T cell IFNγ responses and DNAemia resolution in a small cohort of D+R– kidney transplant patients undergoing primary HCMV infection was observed. Secondly, no correlation between IFNγ production from HCMV peptide stimulated PBMC and the ability of these supernatants to inhibit virus dissemination was observed. Instead, we established that a fully autologous HCMV infection and immune cell co-culture system, incorporating both direct cell-cell and secreted antiviral immunity, was much more effective in distinguishing between HCMV-specific immunity in healthy seropositive and seronegative donors. Using this approach, we demonstrated potent antiviral activity of CD8^+^ T cells and correlated this with control of viraemia in D+R+ transplant recipients.

An important aspect of our study was the increased choice of antigens used to stimulate PBMC. This allowed us to test whether the variable predictive power of IFNγ-focused CMI assays was due to the limited choice of HCMV antigens used for stimulation of T cells. Many commercial assays which enumerate IFNγ responses to HCMV (e.g., T.Track CMV and T.SPOT-CMV) focus on a narrow range of lytic antigens, such as IE1 and pp65. However a number of studies have now demonstrated that the CD4^+^ and CD8^+^ T cell responses to HCMV are broader than this, and that not all seropositive donors will respond to IE1/pp65 (Sylwester et al., 2005; Jackson et al., 2014, 2017a). Some studies have suggested that serology alone may over-estimate the proportion of healthy individuals who are HCMV seropositive compared to testing for HCMV serostatus by Western blot (Sukdolak et al., 2013). We do not believe that false-positive HCMV serology explains all cases of individuals who fail to make a detectable IFNγ response to pp65, as we and others have noted that serostatus cannot be accurately predicted by the CD4^+^ or CD8^+^ T cell response to a single ORF, but rather by the combination of detectable responses to several ORFs (Sylwester et al., 2005; Jackson et al., 2017b). By increasing the range of immunodominant peptides used for CD3^+^ T cell stimulation beyond that used in commercial assays, we could better enumerate IFNγ FluoroSpot responses in seven D+R– SOT recipients. Importantly, this approach still did not identify any obvious differences in the immune responses of these patients to provide an explanation for why some recipients had resolution of viraemia, and others recurrent viraemia despite a broad T cell response. We considered delayed immune reconstitution and immune suppression as factors in the occurrence and recurrence of viraemia, as HCMV-specific CMI is known to reconstitute more quickly in R+ than R– transplant recipients (Eid et al., 2010) and to reconstitute at different rates in different patients (Abate et al., 2010). However, that does not seem to explain the differences between patients who did and did not control viraemia, as all patients mounted T cell responses similar in magnitude and breadth to those seen in healthy seropositive donors. Additionally, all patients received similar immunosuppressive regimes, suggesting other factors might explain the variable efficacy of their T cells.

A major aim of this study was to develop a robust method to identify and assess antiviral CMI responses *in vitro* to empower our ongoing work aimed at understanding the key aspects of the immune response that provide protection in some, but not all, transplant patients. It is well-established that HCMV-infected cell lysates or peptide stimulation can be used to generate IFNγ responses from PBMC and CD4/8^+^ T cells (e.g., Sinclair et al., 2004; Jackson et al., 2017b; Chanouzas et al., 2018). However, it became clear that this form of stimulation did not always translate directly into effective control of viral replication in our assays. Furthermore, they again confirmed that antiviral activity did not correlate with ability of T cells to produce IFNγ. An obvious interpretation is that other cytokines play an important role in the control of viral replication. It is interesting to note that anti-HCMV activity of monocyte derived macrophages has been observed and that this was not mediated by IFNγ nor primary IFNα or IFNβ although the identity of the secreted factor responsible for the activity was not determined (Becker et al., 2018). Other studies have shown that even in the presence of IFNγ or IFNα or IFNβ neutralizing antibodies, HCMV infection can still be controlled by lymphocytes in a non-cytotoxic manner, with granzymes implicated in control (Shan et al., 2020). A non-biased mass spectrometry-based approach is likely to be required to elucidate the key antiviral components of effective secretomes. Taken together we feel that an antiviral assay system based on stimulation of PBMC with HCMV antigens did not provide sufficient specificity and sensitivity as a viable technique to better measure effective CMI in the transplant setting.

To address this, we developed a fully autologous co-culture system to determine anti-HCMV activity of PBMC and/or isolated lymphocyte subsets. Without the need to use individual cytokines as proxies for potential *in vivo* control we reasoned it could provide a much more direct measure of anti-HCMV status of a transplant patient's immune response. One important aspect of our approach is that these responses can be characterized in aggregate (PBMC) and studied at the individual lymphocyte population level (NK or CD8^+^ T cells). As such the approach could potentially inform us about the relative importance of individual lymphocyte subsets and the interplay between these (as seen when PBMC is utilized) in order to mediate effective antiviral responses. Whole PBMC include innate, adaptive and so-called “innately adaptive” immune cells (Ferreira, 2013), which have both individual and potentially synergistic antiviral activity. The antiviral capabilities of PBMC would include directly cytotoxic effector function, and secretion of antiviral soluble factors. In addition, the use of a dual-color fluorescent HCMV strain allows the differential quantification of whether control acts on virus spread or late viral gene expression, or both. As such, the inhibition of viral dissemination demonstrated by PBMC from healthy seronegatives at higher E:T ratios was to be expected, as innate immune cells are present. However, the antiviral activity is rapidly diluted, and this is consistent with a degree of innate cell-mediated (NK cell) control of HCMV spread in both HCMV seropositives and seronegatives that we tested. It was however interesting to observe that NK cells derived from HCMV seropositive donors were no better or worse at controlling HCMV and that individuals with NK cells with good or poor antiviral control could be observed in both groups. We have previously shown that HCMV-specific memory T cells are not present in HCMV seronegative PBMC (Sylwester et al., 2005; Jackson et al., 2017b), which also explains why antiviral activity of PBMC from seronegative donors dilutes rapidly. Consequently, it is not surprising that very clear differences in the antiviral activity of CD8^+^ T cells can be seen between HCMV seropositive and seronegative donors reflecting the fundamental difference in efficacy of primary and memory T cell responses.

Another major advantage of determining antiviral capacity in this viral dissemination co-culture system, is the use of a low passage strain of HCMV that expresses the full complement of immune evasion genes. As such viral derived antigens, both incoming from initial virus infection and produced *de novo*, are processed and presented in the correct temporal and immune evasion contexts. This being the case, it is clear that CD8^+^ T cells derived from seropositive donors are still able to exert antiviral effector function despite immunoevasins (e.g., US2,3,6,11) that interfere with MHC Class I processing and cell surface expression (Reddehase, 2002; Wills et al., 2015). Indeed, this is consistent with HCMV being asymptomatic in healthy individuals due to effective immune control. What this may reflect is that incoming HCMV proteins are processed and presented for T cell recognition prior to the virus interfering with this process, as has been suggested for Epstein-Barr virus (Forrest et al., 2018). It should also be noted that the ability of some HCMV immunoevasins to prevent MHC class I processing and presentation is dependent on the genotype of the host [e.g., US2 cannot bind HLA-B^*^07 and ^*^27 as efficiently as HLA-A genotypes (Gewurz et al., 2001; Reddehase, 2002); US11 degrades HLA-A but is less effective against HLA-B, with HLA-B^*^44:02 particularly resistant (Zimmermann et al., 2019)]. Thus, the HLA genotype of the donor will contribute to the effectiveness of HCMV evasion of CD8^+^ T cell control.

The nature of the responding HCMV specific T cells is also an important factor. It is recognized that CD8^+^ T cells with a high avidity for HCMV peptides would require far less stimulation with their cognate antigens than low avidity CD8^+^ T cells (Villacres et al., 2003), and have potent cytotoxicity, IFNγ production and proliferation in response to HCMV antigen stimulation (Villacres et al., 2003; Ogonek et al., 2017). It has been shown that only a relatively small amount of cell surface MHC class I expression is required to trigger a cytotoxic T cell response to e.g., IE1 further supporting the hypothesis that even minimal MHC class I presentation (despite viral MHC class I immunoevasins) of HCMV antigens to seropositive donor CD8^+^ T cells is sufficient for a strong antiviral response to infected fibroblasts (Besold et al., 2009). High affinity identical or near-identical public TCR sequences arise convergently in unrelated individuals in response to HCMV infection, via antigen-driven selection (Gras et al., 2009). These high-affinity TCRs are part of an evolving T cell response to HCMV antigens in seropositive donors during long term carriage and reactivation events (Schober et al., 2018). More broadly, these observations are again consistent with the concept that measurement of the quality of an immune response is more important than quantity.

During the process of establishing this assay, our studies suggest that there is considerable variability in the ability of different donors' NK cells to control HCMV spread and late gene expression. Furthermore, the data also suggest that no statistically significant difference in the ability of NK cells isolated from seropositives and seronegatives to control HCMV dissemination is evident. This observation is supported by a previous publication studying an independent cohort of NK cell donors, using non-autologous indicator fibroblasts (Chen et al., 2016), and work by other groups showing that not all NK cell clones are equally effective at lysing HCMV-infected autologous fibroblasts (Carr et al., 2002). The ability of NK cells to inhibit late viral gene expression, compared to inhibition of virus spread, is also similar to results from Wu et al. (2015) who observed reduced HCMV UL86 (major capsid protein) expression in NK-fibroblast co-cultures, even though IE antigen expression was seen in almost every cell.

In this study, we did not explore the phenotypic characteristics of the NK cells present in each donor, but it has been shown that there are a number of different NK cell subsets within an individual, independently expressing activating and inhibitory receptors (Cooper et al., 2001). HCMV may further epigenetically reprogram and diversify NK cell function and receptor expression in some subsets (Lee et al., 2015; Schlums et al., 2015). LIR1^+^ and LIR1^−^ NK cells have also been shown to have differential activity against HCMV strains in a viral dissemination assay, suggesting interactions with specific residues within UL18 (Chen et al., 2016). The differences in NK cell control between donors may also reflect a number of host factors (Patel et al., 2018), including NKG2C copy number (Muntasell et al., 2013), HLA/KIR genotype interactions (Hadaya et al., 2008), other host genetic factors (Yu et al., 2018), age (Manser and Uhrberg, 2016), and underlying health conditions (Brunetta et al., 2010). For seropositives, it may also be influenced by the HCMV strain to which the donor has previously been exposed (Chen et al., 2016) and how long ago the donor was infected (Vieira Braga et al., 2015). HCMV also has a repertoire of immunoevasins which impair or reduce NK cell activation (De Pelsmaeker et al., 2018). It is likely that the balance between evasion of NK cell control by the virus and superior NK cell recognition by some donors due to host factors leads to the complex variation in control of virus spread and late gene expression seen in our healthy donor cohorts. Importantly, our assay system provides the framework to systematically investigate these different variables in future studies.

In this study, we have defined a functional assay that measures HCMV cell-mediated immunity and established the baseline antiviral CMI of healthy HCMV seropositives and seronegatives. This assay is highly tractable and will allow the systematic investigation of host and viral factors that influence the ability of clinically relevant patient populations to control (or not control) HCMV viraemia. Importantly, this assay relies on a measure of antiviral activity, not T cell activation. The implications of this are obvious: for example, individuals infected with multiple HCMV strains (e.g., D+R+ cohort) may control their own virus but not the infection with a new strain as effectively. Given that it is likely that the R+ individuals' T cells would likely recognize conserved HCMV antigens from either strain we can now ask how this translates into the control of the replication of multiple strains of HCMV by combining this assay with the power of whole viral genome sequencing and HCMV BAC recombineering. It is anticipated that the integration of these approaches will shed new light on the immune parameters and mechanisms critical for the control of HCMV *in vivo*.

## Data Availability Statement

All datasets generated for this study are included in the article/Supplementary Material.

## Ethics Statement

This work was reviewed and approved by the Medical Ethics Committee of the Academic Medical Centre, Amsterdam; the London—Queen Square Research Ethics Committee (REC reference 17/LO/0916); the Cambridge Central Research Ethics Committee (97/092); the Cambridge Human Biology Research Ethics Committee (HBREC.2014.07); and the North of Scotland Research Ethics Committee 1 (NS/17/0110). In each case, informed written consent was obtained from all volunteers in accordance with the Declaration of Helsinki.

## Author Contributions

SJ, CH, EL, and MW designed the study. CH, SJ, MR, and MW wrote the manuscript. CH, SJ, EL, GS, ED, and MW performed the experimental work. CH, SJ, EL, GS, and MW analyzed the data. CA, MM, ER, GO, FB, and RS contributed to experiments. MW supervised the study. All authors read and approved the final manuscript.

## Conflict of Interest

The authors declare that the research was conducted in the absence of any commercial or financial relationships that could be construed as a potential conflict of interest.
