# Supplementary material for: Assessing Anti-HCMV Cell Mediated Immune Responses in Transplant Recipients and Healthy Controls Using a Novel Functional Assay
Source: Front Cell Infect Microbiol. 2020 Jun 26;10:275. doi: 10.3389/fcimb.2020.00275 (PMC7332694; doi:10.3389/fcimb.2020.00275)
Supplement: Supplementary file 1 [file Data_Sheet_1.docx]

Article Title: Assessing anti-HCMV cell mediated immune responses in transplant recipients and healthy controls using a novel functional assay

Supplementary table 1: AMC patient characteristics

| Patient ID | Age at Tx | Immuno-suppressive therapy | Proph-ylaxis | Antiviral therapy | First detection of virus (week post Tx) | First peak viraemia (week post Tx) | CMV IgG+ (weeks post Tx) | Last sample (week post tx) |
| --- | --- | --- | --- | --- | --- | --- | --- | --- |
| 133 | 66 | P/CsA/MMF | No | No | 8.7 | 12.14 | 15.71 | 123.3 |
| 136 | 31 | P/CsA/MMF | No | Yes | 6.6 | 8.57 | 10.57 | 78.6 |
| 197 | 31 | P/MMF/FK/Cd25mAb | No | No | 14.0 | 14.00 | 14.00 | 157.9 |
| 352 | 26 | P/CsA/CD25mAb/MMF | No | Yes | 4.9 | 7.14 | 7.14 | 97.4 |
| 365 | 54 | P/CsA/CD25mAb/MMF | No | Yes | 6.9 | 6.86 | 8.57 | 116.7 |
| 439 | 21 | P/MMF/FK/Cd25mAb | No | Yes | 6.6 | 9.00 | 10.00 | 16.6 |
| 574 | 50 | P/MMF/FK/Cd25mAb | Months 1-3, 6-9 | No | 6.1 | 6.29 | 25.43 | 37.3 |

Immunosuppressives key:

P – Prednisone

CsA – Cyclosporin

FK – Tacrolimus

MMF – mycophenolate mofetil

Supplementary table 2: UCL D+R+ patient charactistics

| **Patient ID** | **Age** | **Transplant status** |
| --- | --- | --- |
| R02-00005 | 53 | D+R+ |
| R02-00058 | 46 | D+R+ |
| R02-00079 | 29 | D+R+ |
| R02-00109 | 54 | D+R+ |

Supplementary Table 3: Primer sequences for construction of Merlin mCherry-P2A-UL36 GFP-UL32

| UL32-rpsl-F | CTCCGTCCGTCCTCCTTTCCCGACACGTCACTATCCGATGATTTCATTAAAAAGTACGTCTGCGTGTGTGTTTCTTAACCTGTGACGGAAGATCACTTCG |
| --- | --- |
| UL32-rpsl-R | CATGGGGGGCGCCAAAACGCCGTCGGACGCCGTGCAGAACATCCTCCAAAAGATCGAGAAGATTAAGAACACGGAGGAACTGAGGTTCTTATGGCTCTTG |
| UL32-eGFP-F | CGGACGCCGTGCAGAACATCCTGCAAAAGATCGAGAAGATTAAGAACACGGAGGAAGGAAGCGGAGGTTCTGGTGGATCTGGAATGGTGAGCAAGGGCGA |
| UL32-eGFP-R | CTCCGTCCGTCCTCCTTTCCCGACACGTCACTATCCGATGATTTCATTAAAAAGTACGTCTGCGTGTGTGTTTCTTAATTACTTGTACAGCTCGTCCATG |
| UL36-rpsl-F | TTATATAAAATGCTGTGTTATATACAAAAACATGCACATAGACAGACAGAACCACCGTGCTCGTCGTCCCCTCCTTAACCTGTGACGGAAGATCACTTCG |
| UL36-rpsl-R | CGGTCGACGGCACATTATTCCCGGCGCCGCCAACGGCATGCCGCCCCTCACCCCGCCACACGCCTACATGAACAACTGACTGAGGTTCTTATGGCTCTTG |
