# Supplementary material for: Assessing Anti-HCMV Cell Mediated Immune Responses in Transplant Recipients and Healthy Controls Using a Novel Functional Assay
Source: Front Cell Infect Microbiol. 2020 Jun 26;10:275. doi: 10.3389/fcimb.2020.00275 (PMC7332694; doi:10.3389/fcimb.2020.00275)
Supplement: Supplementary file 2 [file Data_Sheet_2.docx]

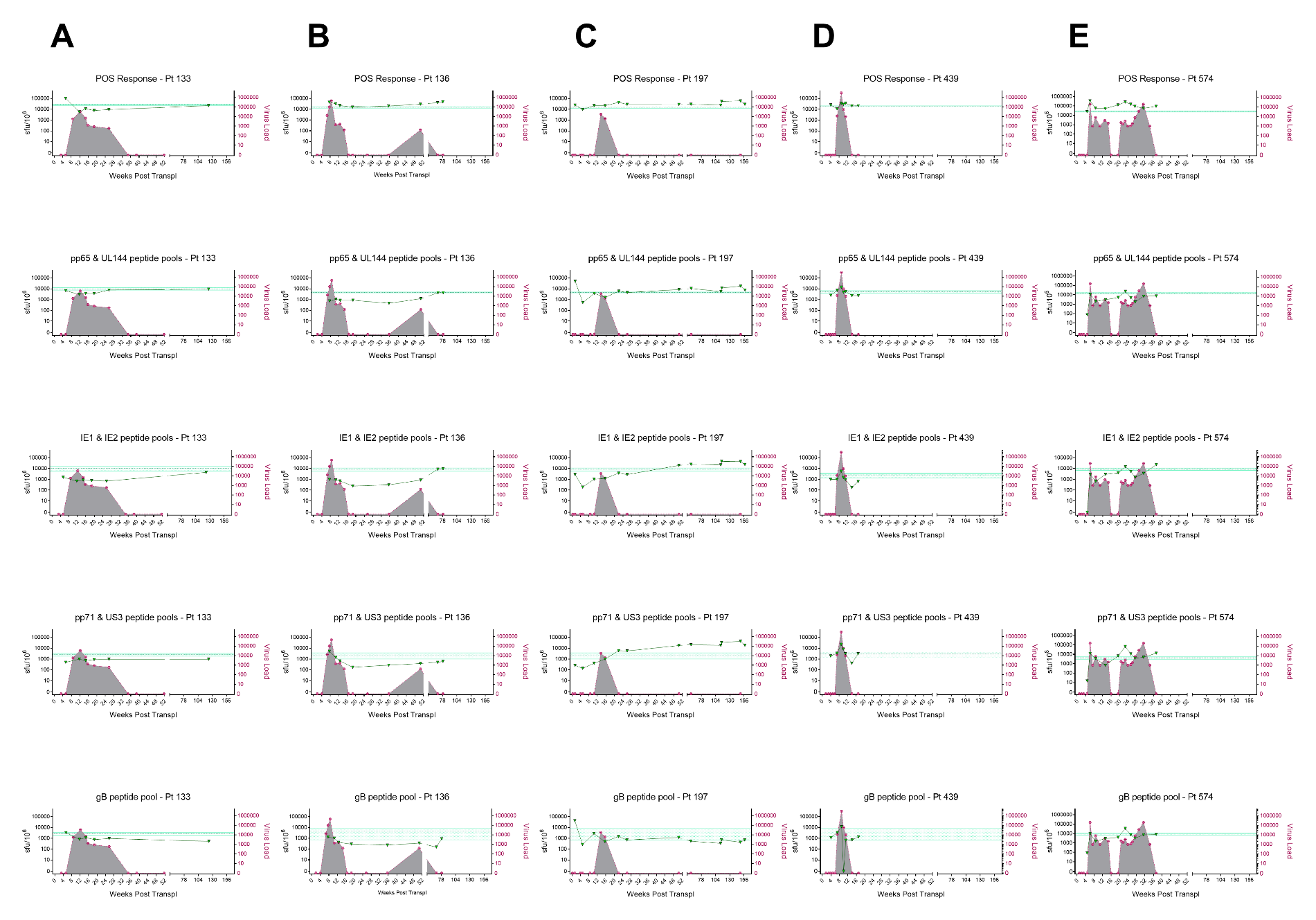


**Supplementary figure 1**

**Analysis of longitudinal HCMV virus load and HCMV-specific CD3^+^ T cell IFNy responses of** **D+R- kidney transplant patients.**

Five D+R- kidney patients with primary HCMV infection, T cell responses (spot forming units per 10^6 CD3+ T cells) were measured by IFNγ FluoroSpot (green triangles connected by a solid line) to HCMV peptide pools covering covering pp65 and UL144, IE1 and IE2, pp71 and US3, and gB, as well as polyclonal T cell stimulation as a positive control (‘POS’). Virus load (copies/ml blood) was measured by QNAT of HCMV DNA (pink hexagons connected by a dashed line). Cyan lines show the mean magnitude of response (+/- standard error) of CD3^+^ T cell IFNγ responses seen in healthy seropositive individuals in the same age decade as the transplant recipient for each peptide pool (Jackson, 2017b).

**
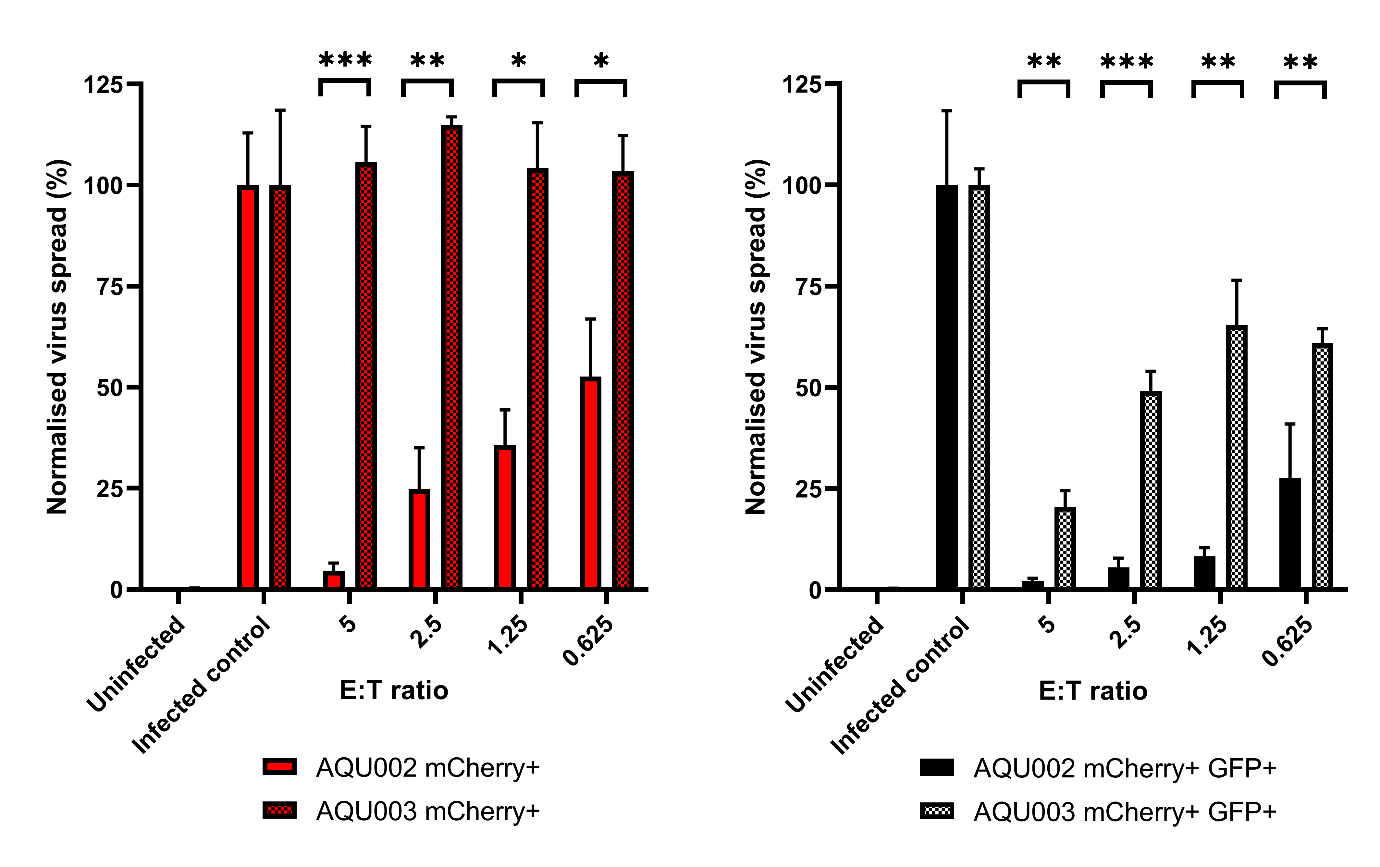
**

**Supp figure 2**

**Analysis of HCMV restriction by NK cells derived from two age-matched seropositive donors**

The effectiveness of NK cells from two age-matched HCMV seropositive donors in an autologous virus dissemination assay. Filled bars are donor AQUARIA002; hatched bars are donor AQUARIA003. NK cells derived from donor AQUARIA002 were more effective at inhibiting virus dissemination than NK cells derived from donor AQUARIA003 across a range of E:T ratios (one-tailed T test, p < 0.05).


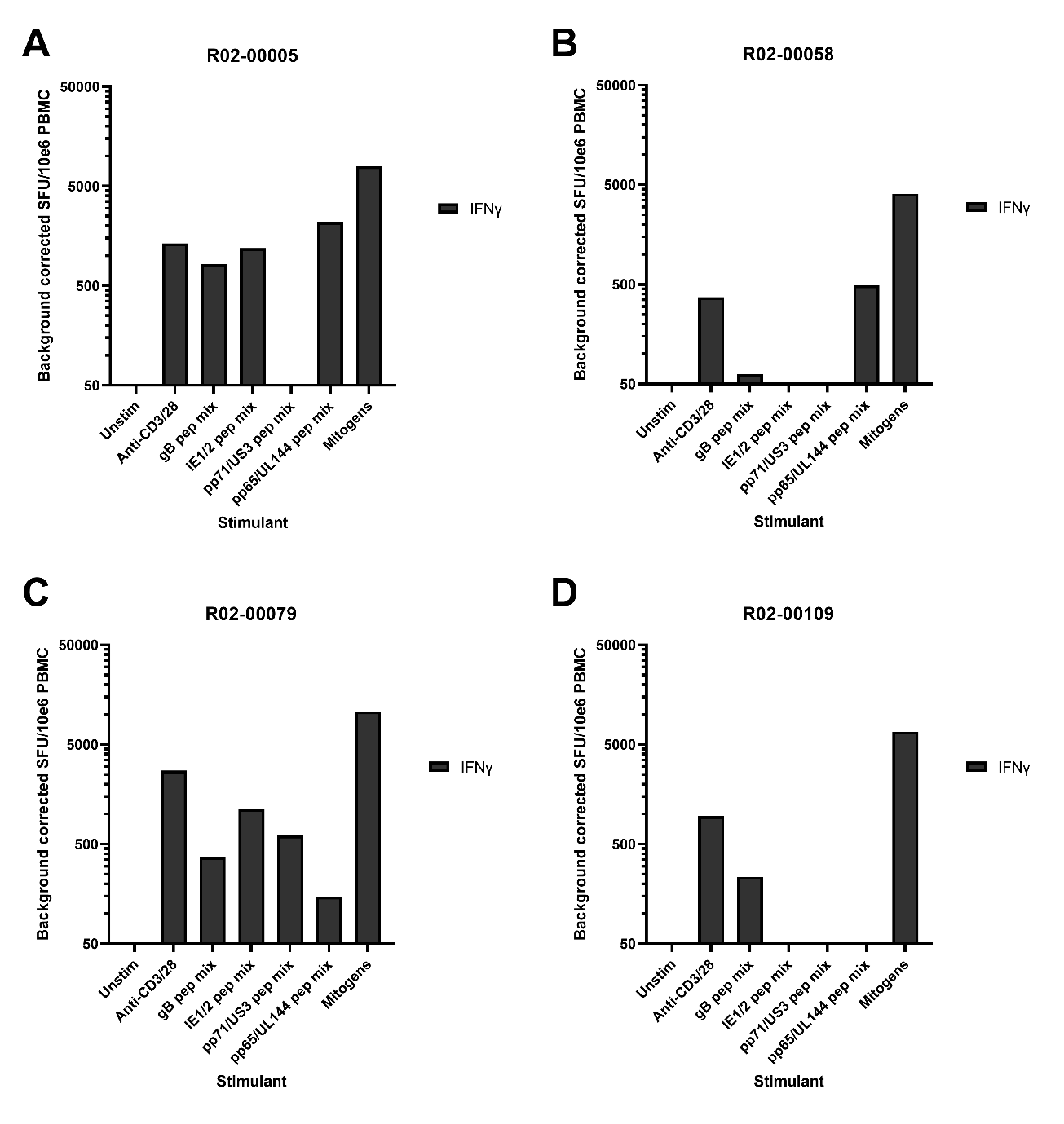


**Supplementary figure 3**

**Analysis of HCMV specific IFNγ FluoroSpot responses to HCMV peptide pools in** **non-viraemic kidney transplant recipients**

IFNγ FluoroSpot responses to HCMV peptide pools covering gB, IE1 and IE2, pp71 and US3, and pp65 and UL144, as well as polyclonal anti-CD3/28 antibody T cell stimulation as a positive control, from four D+R+ non-viraemic kidney transplant recipients approximately three months post-transplant. FluoroSpot responses are calculated as spot-forming units (SFU) per 10^6 PBMC (background corrected).
